# Adjudin-preconditioned neural stem cells enhance neuroprotection after ischemia reperfusion in mice

**DOI:** 10.1186/s13287-017-0677-0

**Published:** 2017-11-07

**Authors:** Tingting Zhang, Xiao Yang, Tengyuan Liu, Jiaxiang Shao, Ningzhen Fu, Aijuan Yan, Keyi Geng, Weiliang Xia

**Affiliations:** 1 School of Biomedical Engineering & Med-X Research Institute, Shanghai Jiao Tong University, Shanghai, China; 2 Department of Neurology & Institute of Neurology, Rui Jin Hospital, School of Medicine, Shanghai Jiao Tong University, Room 211, Med-X Research Institute, 1954 Huashan Road, Shanghai, 200030 China

**Keywords:** Stroke, Neural stem cells, Adjudin, Inflammation, Oxidative stress, Blood–brain barrier

## Abstract

**Background:**

Transplantation of neural stem cells (NSCs) has been proposed as a promising therapeutic strategy for the treatment of ischemia/reperfusion (I/R)-induced brain injury. However, existing evidence has also challenged this therapy on its limitations, such as the difficulty for stem cells to survive after transplantation due to the unfavorable microenvironment in the ischemic brain. Herein, we have investigated whether preconditioning of NSCs with adjudin, a small molecule compound, could enhance their survivability and further improve the therapeutic effect for NSC-based stroke therapy.

**Method:**

We aimed to examine the effect of adjudin pretreatment on NSCs by measuring a panel of parameters after their transplantation into the infarct area of ipsilateral striatum 24 hours after I/R in mice.

**Results:**

We found that pretreatment of NSCs with adjudin could enhance the viability of NSCs after their transplantation into the stroke-induced infarct area. Compared with the untreated NSC group, the adjudin-preconditioned group showed decreased infarct volume and neurobehavioral deficiency through ameliorating blood–brain barrier disruption and promoting the expression and secretion of brain-derived neurotrophic factor. We also employed H_2_O_2_-induced cell death model in vitro and found that adjudin preconditioning could promote NSC survival through inhibition of oxidative stress and activation of Akt signaling pathway.

**Conclusion:**

This study showed that adjudin could be used to precondition NSCs to enhance their survivability and improve recovery in the stroke model, unveiling the value of adjudin for stem cell-based stroke therapy.

**Electronic supplementary material:**

The online version of this article (doi:10.1186/s13287-017-0677-0) contains supplementary material, which is available to authorized users.

## Background

Ischemic stroke represents the most common cause of serious morbidity and mortality, which is the second major cause of disability worldwide [1]. Few pharmacotherapies have drawn the attention of medical circles and one therapy is the recanalization of occluded vessels via thrombolysis using tissue plasminogen activator (tPA), which due to a narrow time window can only be applied to a minority of patients [2, 3]. Because of limitations and complications in tPA-based treatment, restorative therapies are urgently needed to promote brain remodeling and repair once acute ischemic stroke (AIS) injury has occurred. Fortunately, stem cell-based strategies have emerged as a promising therapeutic strategy for AIS and gained increasing interest in recent years for their unique properties of action, which is the ability to abrogate subacute and chronic secondary cell death associated with the disease [4, 5]. Currently, different types of stem cells are used for the treatment of ischemic stroke including neural stem cells (NSCs) [6], mesenchymal stem cells (MSCs) [7], oligodendrocyte progenitor cells (OPCs) [8], embryonic stem cells (ESCs) [9], endothelial progenitor cells (EPCs) [10, 11], induced pluripotent stem cells (iPSCs) [12], vascular progenitor cells (VPCs) [7], and so forth. These stem cells could secrete various neurotrophic factors and cytokines, or differentiate into multiple cell types to compensate for I/R-induced cell death, strengthen the connection between the synapses, and establish new neural circuits to attenuate ischemic brain injury and finally improve neurobehavioral recovery [13, 14]. And in the clinical study, a number of preliminary trials found that transplanting stem cells to patients between 3 days and 24 months after stroke was feasible and safe [15, 16]. However, recent evidence consistently challenges this therapy on its limitations, especially the hostile microenvironment in the ischemic brain which presents a significant hurdle to the survival of transplanted cells. Hicks et al. [17] demonstrated that only 1–3% of grafted cells survived in the ischemic brain 28 days after grafting. The massive death of transplanted stem cells will hamper the application of cell-based therapy, which might be influenced by the production of reactive oxygen species (ROS) and inflammatory response mediators after I/R injury [18–20]. Thus finding a strategy to overcome this obstacle would potentially be of great value.

In order to resolve the problem of cell survival after transplantation, several remedial approaches have been suggested. Both preconditioned stem cells and gene modification exhibited an improvement of cell viability after transplantation [21–24]. However, although these methods showed a better transplantation outcome, some challenges still existed in using chemical factors to precondition stem cells or through modifying certain genes in stem cells. For example, lipopolysaccharide (LPS), IL-6, minocycline, and melatonin were all available factors for stem cell preconditioning, which could reduce cell death, increase stem cell proliferation and neurotrophic factor secretion, enhance cytoprotection and angiogenesis, and accelerate functional recovery in acute and subacute ischemia [25–28]. However, LPS could cause neuroinflammation, hypotension, or sepsis in pathological injury [29]; IL-6-pretreated MSCs could promote osteosarcoma growth, which suggested that IL-6 mediated the recruitment of MSCs to facilitate tumor progression [30]. So far, it has been considered that minocycline and melatonin have low-toxicity and they are biologically natural agents which were used to pretreatment cells. As for gene modification, uncontrolled expression of introduced genes could cause many adverse impacts on the body, such as leukemia, which has been attributed to insertional mutagenesis that combined with acquired somatic mutations following gene therapy of SCID-X1 patients [31]. Compared with gene modification, preconditioned stem cell therapy seemed more beneficial, simpler, and safer for ischemic stroke therapy [32]. Therefore, we wish to offer safe and effective drugs which could combine with NSCs for future clinical application.

Adjudin, 1-(2,4-dichlorobenzyl)-1H-indazole-3-carbohydrazide, formerly called AF-2364, is a reversible antispermatogenic compound, which is under development as a potential nonhormonal male contraceptive that can disrupt the adherens junction of germ cells to Sertoli cells without affecting testosterone production [33]. Adjudin is a small molecular derivative of indazole and is an analog of the chemotherapy drug lonidamine which had been demonstrated to have no apparent side effects in treated animals [33]. It has also been reported that many indazole derivatives are nonsteroidal anti-inflammatory drugs (NSAID) which could suppress the production of prostaglandin E2 (PGE2) synthesis and nitric oxide (NO) and the release of cytokines and chemokines [34]. Our previous results demonstrated that adjudin could protect against cerebral I/R injury by inhibition of neuroinflammation and blood–brain barrier (BBB) disruption through intraperitoneal injection [35]. We also found that adjudin could attenuate LPS-induced BV2 activation by suppression of the NF-κB pathway [36], which showed that adjudin appears to be a promising neuroprotective agent for ischemic stroke therapy. In this study, we aimed to examine whether adjudin pretreatment on NSCs could have a better effect on neuroprotection compared with nonpreconditioned NSCs after I/R injury.

## Methods

### Cell culture and characterization

All animal experimental protocols were approved by the Institutional Animal Care and Use Committee (IACUC) of Shanghai Jiao Tong University, Shanghai, China (Permission number: Bioethics 2012022). NSCs were harvested from the cortex of the E14 green fluorescent protein (GFP)-transgenic mice (Animal Research Center of Nanjing University, Nanjing, China). In brief, bilateral cortex zones from mouse brains were dissected in HBSS and dissociated mechanically. The cells were collected and resuspended in DMEM/F12 (1:1) medium (Gibco, Carlsbad, CA, USA) containing B27 supplement (Gibco), l-glutamine (Sigma-Aldrich), 20 ng/ml mouse basic fibroblast growth factor (Gibco), and 20 ng/ml mouse epidermal growth factor (Gibco). Cells were monolayer cultured on a 60-mm plastic dish (Corning Incorporated, Corning, NY, USA) precoated with poly-l-ornithine hydrobromide (Sigma, St Louis, MO, USA) and laminin (Sigma) at 37 °C with 5% CO_2_ in an incubator (Thermo Scientific, Barrington, IL, USA). The medium was changed every 2 days and cells were passaged in about 5 days. Cells that had been passaged three to five times were used for the experiments, which strongly maintained their proliferation and differentiation ability.

To characterize cells, NSCs were cultured on poly-l-ornithine hydrobromide (Sigma) and laminin (Sigma)-coated glass coverslips in a 24-well plate (Corning). Cells were then immunostained with mouse anti-Nestin (1:200; Millipore, Billerica, MA, USA), goat anti-Sox2 (1:100; Santa Cruz Technology, Santa Cruz, CA, USA), rabbit anti-glial fibrillary acidic protein (GFAP) (1:200; Millipore), and mouse anti-Doublecortin (1:100; Santa Cruz Technology).

### Adjudin pretreatment of NSCs

The NSCs were preconditioned with adjudin before the in-vitro experiments or transplantation. Adjudin was added to the cell culture medium with a final concentration of 0, 5, 10, 30, or 60 μM for 24 hours, followed by drug washout before experiments. Cell death was quantified by a standard measurement of lactate dehydrogenase (LDH) release assay as described previously [36]. Cell viability was assessed by CCK-8 assay kit (Dojindo Laboratories, Kumamoto, Japan). Data were acquired using a microplate reader (Synergy2; BioTek, Winooski, VT, USA).

### Cell death and cell survival analysis in vitro

To evaluate NSC viability under oxidative stress, NSCs were seeded at a density of 1 × 10^5^ or 1 × 10^4^ cells per well in 24-well culture plates or 96-well culture plates (Corning) respectively and subjected to different concentrations of H_2_O_2_ (0.05, 0.1, 0.3, 0.5 mM; Sigma) for 1 hour. NSCs were then washed three times with phosphate-buffered saline (PBS) and cultured for another 24 hours in high-glucose DMEM with 10% FBS. These NSCs were then examined by LDH assay and CCK-8 assay kit.

To determine the effect of adjudin on NSC viability under oxidative stress, NSCs were pretreated by adjudin with the concentration of 10 or 30 μM for 24 hours. The cells were then washed three times with PBS and subjected to 0.1 mM H_2_O_2_ for 1 hour followed by LDH assay and CCK-8 assay 24 hours later.

### ATP assay

ATP levels were quantified using the Roche ATP Bioluminescence Assay Kit (HS II; Indianapolis, IN, USA) following the standard protocol provided by the vendor. In brief, cells were washed once with PBS and lysed with the Cell Lysis Reagent for 15 min. Then 50 μl of the homogenates were mixed with 150 μl of the Luciferase Reagent, and the luminescence was detected using a microplate reader (Synergy2). The protein concentrations of the samples were quantified with bicinchoninic acid (BCA) protein assay (Pierce, Rockford, IL, USA). The ATP concentrations of the sample were calculated using an ATP standard, and normalized against the protein of the samples.

### Cell proliferation and differentiation in vitro

To evaluate NSC proliferation and differentiation after treatment with adjudin in vitro, NSCs were monolayer cultured on poly-l-ornithine hydrobromide (Sigma) and laminin (Sigma)-coated glass cover slips in a 24-well plate (Corning). After pretreatment with adjudin at concentrations of 10 or 30 μM for 24 hours, NSCs were washed with fresh medium to remove the drug. Then 3 days later, cells were immunostained with mouse anti-Nestin (Millipore), goat anti-Sox2 (Santa Cruz Technology), rabbit anti-glial fibrillary acidic protein (GFAP) (Millipore), mouse anti-Doublecortin (Santa Cruz Technology), and rabbit anti-Ki67 (1:200; Abcam, Cambridge, MA, USA).

### Transient middle cerebral artery occlusion model

Focal cerebral ischemia in mice was performed as described previously [35]. In brief, adult male ICR mice weighing 25–30 g were anesthetized with ketamine/xylazine (100 mg/10 mg/kg; Sigma) intraperitoneally. Body temperature was maintained at 37 ± 0.5 °C using a heating pad (RWD Life Science, Shenzhen, China). Under the surgical microscope (Leica, Solms, Germany), the left common carotid artery (CCA), the external carotid artery (ECA), and the internal carotid artery (ICA) were isolated. Then a 6-0 suture (Dermalon, 1741-11; Covidien, OH, USA) with a round tip and coated with silicone was inserted from the ECA into the ICA and reached the circle of Willis to occlude the origin of the middle cerebral artery (MCA) until a slight resistance was felt. The distance from the furcation of the ECA/ICA to the opening of the MCA was 9 ± 0.5 mm. The success of occlusion was determined by monitoring the decrease in surface cerebral blood flow to 80% of baseline, which was verified by a laser Doppler flow-meter (Moor LAB; Moor Instruments, Devon, UK). Reperfusion was performed by withdrawing the suture 2 hours after middle cerebral artery occlusion (MCAO). To confirm successful occlusion/reperfusion, cerebral blood flow was tested again. The sham operated mice were subjected to the same procedure except for the suture insertion.

### NSC transplantation

Twenty-four hours after transient middle cerebral artery occlusion (tMCAO), mice were divided randomly into three groups for NSC or vehicle injection: PBS group, NSC group, and adjudin-pretreated group. The animals were anesthetized with ketamine/xylazine intraperitoneally, and received stereotaxic transplantation. Adjudin-pretreated or untreated NSC suspension with 1 × 10^6^ cells in 5–15 μl PBS was injected into the striatum of the ipsilateral hemisphere in mice, with the following coordinates: M–L, −1.5 mm; D–V, −3.25 mm. The same amount of PBS was injected as control. Deposits were delivered at 0.5 μl/min and the needle was left in situ for 5 min post injection before being removed slowly. The wound was then closed and the animal was returned to the cage for follow-up experiments.

### Behavioral assessment

Three days after tMCAO, modified neurological severity scores (mNSS) were assessed by an investigator who was blind to the treatment regimen to assess the neurological status of the animals, which is a composite of motor, reflex, and balance tests (normal score, 0; maximal deficit score, 14) as described previously [37]. Total neurological score was calculated as the sum of scores on limb flexion (range 0–3), walking gait (range 0–3), beam balance (range 0–6), and reflex absence (range 0–2).

The rotarod test required mice to balance on a rotating rod. Mice were given 1-min adaption on the rod, which were then accelerated up to 40 rpm within 2 min. The duration of mice remaining on the rotating rod was recorded. Mice were examined at various time points (≤35 days) after NSC transplantation.

### Measurement of infarct volume

Mice from each group were sacrificed 3 days after cell transplantation. Following PBS solution perfusion, mouse brains were perfused with 4% paraformaldehyde (PFA) and brains immediately removed and frozen in prechilled isopentane and stored at −80 °C. The tissues were then cut into a series of 20-μm-thick coronal sections from the beginning of the infarct area to the end, and one section out of every 10 was collected on the same slide to have a representative cerebral injury with the distance between adjacent sections of 200 μm. The entire set of brain sections was immersed in 0.1% cresyl violet (Sinopharm Chemical Reagent Co., Shanghai, China) for 30 min and then rinsed in distilled water for 10 min. The infarct area in each section was calculated using NIH ImageJ software by the following formula:

$$ \mathrm{Infarct}\  \mathrm{area}\ \left({\mathrm{mm}}^2\right)=\mathrm{contralateral}\  \mathrm{hemisphere}\  \mathrm{area}\ \left({\mathrm{mm}}^2\right)\hbox{--} \mathrm{ipsilateral}\  \mathrm{undamaged}\  \mathrm{area}\ \left({\mathrm{mm}}^2\right). $$

The infarct volume between two adjacent sections was calculated by the following equation:

$$ 1/3\times h\left(S1+S2+\sqrt{S{1}^{\ast }S2}\right), $$

where *S*1 and *S*2 are the infarct areas of the two sections and *h* is the distance between them. The total infarct volume was calculated by the sum of all infarct volume from each pair of adjacent sections [38].

### Immunohistological staining

Cultured NSCs or brain sections (20 μm in thickness) were fixed with absolute methanol in a −20 °C freezer for about 10 min and then washed three times in PBS, and the slices were blocked in 10% normal donkey serum (Jackson ImmunoResearch, West Grove, PA, USA) for 30 min at RT. Cryosections were incubated with one of the following primary antibodies in 1% of the blocking serum at 4 °C overnight: mouse anti-CD11b antibody (1:100; BD Biosciences, San Jose, CA, USA), rabbit anti-Occludin (1:100; Invitrogen, Carlsbad, CA, USA), rabbit anti-ZO-1 (1:100; Invitrogen), and goat anti-CD31 antibodies (1:100; R&D Systems, Tustin, CA, USA). After being washed three times with PBS, sections were incubated with Alexa-488-conjugated secondary anti-body (1:500 dilution; Life Technologies, CA, USA) containing 1% normal donkey serum at RT for 1 hour in darkness, and nuclei were stained with 4,6-diamidino-2-phenylindole (DAPI) (1:500 dilution; Beyotime Institute of Biotechnology, China) for 10 min. After washing with PBS, slides were mounted with antifade mounting medium (Beyotime) and images were acquired under a Leica upright microscope (Leica DM2500) or a confocal laser-scanning microscope (Leica TCS SP5 II). IgG detection in the brain parenchyma was used to indicate the integrity of BBB. These brain sections were incubated with donkey anti-mouse IgG conjugated with biotin (1:500; Life Technologies), and visualized by adding with avidin-Alexa Fluor 488.

### Western blot analysis

Tissue samples were collected from the striatum and cortex of the ipsilateral hemisphere, and sheared, briefly processed ultrasonically, and lysed in lysis buffer (Thermo Scientific, Rockford, IL, USA) containing Complete Protease Inhibitor Cocktail, Phosphatase Inhibitor Cocktail, and 2 mM phenylmethylsulfonyl fluoride (PMSF). The lysates were centrifuged at 12,000 rpm for 20 min at 4 °C, and the supernatants were collected. Immunoblotting was carried out as described previously [39]. A BCA assay kit (Pierce) was used for total protein quantification. Total proteins (40 μg) were denatured at 95 °C for 5 min and electrophoresed through 10 or 6% (for ZO-1) SDS-PAGE and then electrotransferred to 0.45-μm nitrocellulose membranes (Whatman, Piscataway, NJ, USA). Membranes were then blocked with 5% skim milk for 1 hour at RT and incubated with primary antibody solutions respectively at 4 °C overnight. After four washes in TBST, the membranes were hybridized with appropriate HRP-conjugated secondary antibody (1:5000; Jackson) for 1 hour at RT and washed four times with TBST again. The final detection was visualized using enhanced chemiluminescence (ECL) (Thermo Scientific, Rockford, IL, USA). Western blotting reagents and images were captured using the ChemiDoc XRS system (BioRad, Hercules, CA, USA). Loading differences were normalized using an anti-actin antibody with 1:1000 dilution (Santa Cruz Biotechnology, Santa Cruz, CA, USA). The primary antibodies used were as follows: p-AKT/AKT (1:2000; Epitomics, Burlingame, CA, USA); p-p38/p-38, p-JNK/JNK, and p-ERK/ERK (1:1000; Cell Signaling Technology, Danvers, USA); iNOS (1:1000; Abcam); catalase and SOD2 (1:1000; Santa Cruz); BDNF (1:500; Bioworld Technology, USA); β-tubulin (1:2000; Sigma); and β-actin (1:1000; Santa Cruz). The intensity analysis was carried out using the Gel-Pro Analyzer (Media Cybernetics, Silver Spring, MD, USA).

### Real-time PCR

Total RNA from NSCs and brain tissue samples was isolated using Trizol Reagent (TaKaRa, Dalian, China). The concentration of RNA was measured by a spectrophotometer (NanDrop1000; Thermo, Wilmington, DE, USA) followed by a reverse transcription process using the PrimeScript RT reagent kit (TaKaRa). Quantitative real-time PCR was performed on ABI 7900HT using SYBR Premix Ex Taq (TaKaRa) and the following primer pairs for different genes. These primers are as follows: iNOS, sense 5′-GTTCTCAGCCCAACAATACAAGA-3′ and anti-sense 5′-GTGGACGGGTCGATGTCAC-3′; catalase, sense 5′-ACGCAATTCACACCTACACG-3′ and anti-sense 5′-TCCAGCGTTGATTACAGGTG-3′; SOD2, sense 5′-GCGGTCTAAACCTCAAT-3′ and anti-sense 5′-TAGGGCTCAGGTTTGTCCAG-3′; IL-6, sense 5′-TAGTCCTTCCTACCCCAATTTCC-3′ and anti-sense 5′-TTGGTCCTTAGCCACTCCTTC-3′; IL-1β, sense 5′-GCAACTGTTCCTGAACTCAACT-3′ and anti-sense 5′-ATCTTTTGGGGCGTCAACT-3′; TNF-α, sense 5′-CCCTCACACTCAGATCATCTTCT-3′ and anti-sense 5′-GCTACGACGTGGGCTACAG-3′; BDNF, sense 5′-TCATACTTCGGTTGCATGAAGG-3′ and anti-sense 5′-AGACCTCTCGAACCTGCCC-3′; NGF, sense 5′-TGATCGGCGTACAGGCAGA-3′ and anti-sense 5′-GCTGAAGTTTAGTCCAGTGGG-3′; GDNF, sense 5′-CCAGTGACTCCAATATGCCTG-3′ and anti-sense 5′-CTCTGCGACCTTTCCCTCTG-3′; Arg-1, sense 5′-GAACACGGCAGTGGCTTTAAC-3′ and anti-sense 5′-TGCTTAGCTCTGTCTGCTTTGC-3′; CD16, sense 5′-TTTGGACACCCAGATGTTTCAG-3′ and anti-sense 5′-GTCTTCCTTGAGCACCTGGATC-3′; and Rplp0, sense 5′-AGATTCGGGATATGCTGTTGGC-3′ and anti-sense 5′-TCGGGTCCTAGACCAGTGTTC-3′. PCR was performed using the following conditions: denaturing at 95 °C for 10 s, followed by 40 cycles of 95 °C for 5 s and 60 °C for 30 s. Data were analyzed using the comparative threshold cycle (Ct) method, and results were expressed as fold difference normalized to Rplp0.

### Evans Blue extravasation

Mice were anesthetized with ketamine/xylazine, and then 4 ml/kg of 2% Evans Blue (Sigma) in normal saline was injected through the left jugular vein at 3 days following tMCAO. After 2 hours of circulation, the mice were anesthetized and perfused with normal saline. The ipsilateral and contralateral hemisphere of the mice were removed and weighed. Then EB was extracted by homogenizing the samples in 1 ml of 50% trichloroacetic acid solution followed by centrifuging at 12,000 rpm for 20 min. The supernatant was diluted with 100% ethanol at a ratio of 1:3. The amount of EB was determined quantitatively by measuring the 610 nm absorbance of the supernatant (BioTek, Winooski, VT, USA).

### CD31/BrdU double immunostaining

Brains were post-fixed for 24 hours followed by 48 hours of immersion in 30% sucrose in PBS and immediately frozen, and then sectioned using a freezing microtome (Leica, Solms, Germany). A thickness of 20-μm coronal sections was cut. Floating coronal sections were collected in antigen protective solution, which includes 20% glycol, 30% glycerol, and 50% PBS. Sections were first treated with 2 mol/L HCl for 20 min at 37 °C and then neutralized with sodium borate twice each for 10 min. Sections were then treated with 0.3% Triton-X 100 in PBS for 15 min, blocked by 10% BSA, and incubated with CD31 (1:200; R&D) and BrdU (1:50; Santa Cruz) antibody at 4 °C overnight. Finally, the sections were incubated with secondary antibodies (1:500; Thermo Fisher) for 60 min at room temperature. Stained sections were mounted after rinsing.

### Statistical analysis

Each experiment was repeated at least three times. All data are presented as mean ± SEM. Data were analyzed by a one-way ANOVA, followed by Tukey’s honest significant test using the GraphPad InStat (GraphPad Software Inc., La Jolla, CA, USA). *P* < 0.05 was considered statistically significant.

## Results

### NSC culture and characterization

**Fig. 1:**
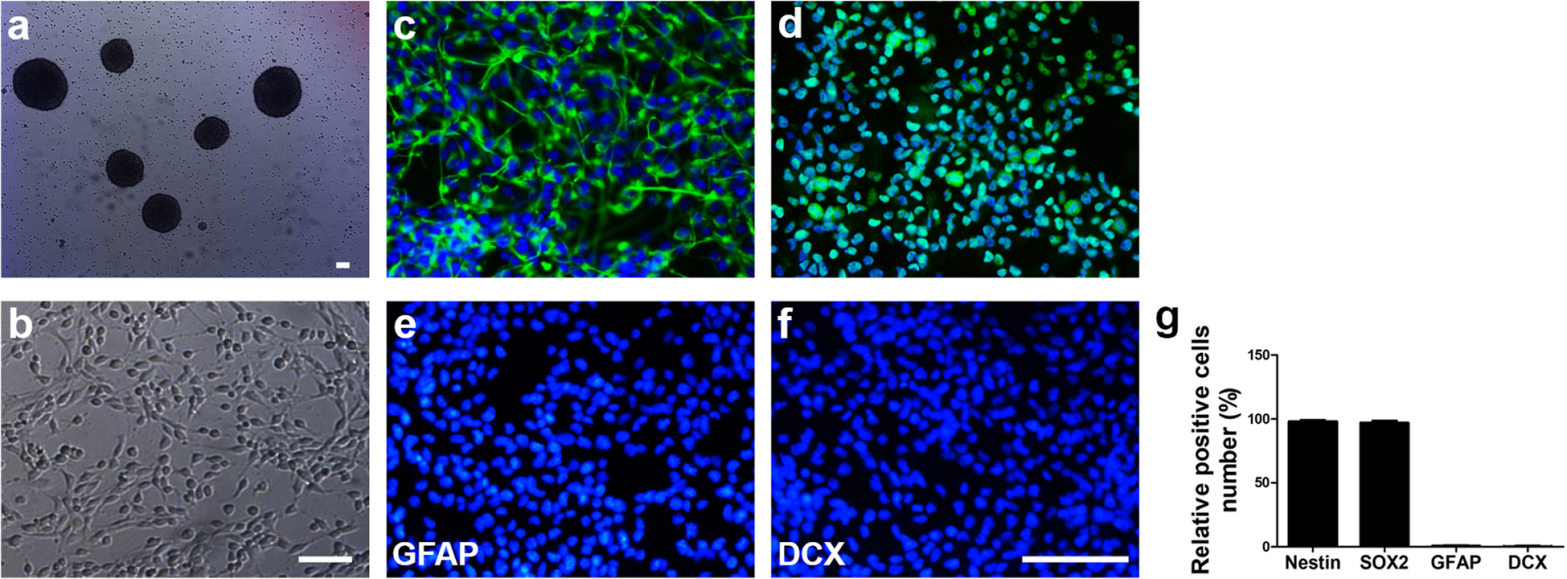
NSC culture and characterization. Morphological analysis of NSCs from mouse cortex. Phase-contrast photomicrographs of suspension neurospheres (**a**) and monolayer culture cells (**b**). Scale bar = 100 μm. Identification of cultured NSCs. Fluorescent photomicrographs of NSCs for Nestin (**c**), SOX2 (**d**), GFAP (**e**), and DCX (**f**). Nuclei were stained with DAPI. Scale bar = 100 μm. Quantifications for GFAP^+^, Nestin^+^, SOX2^+^, and DCX^+^ cells (**g**)

Neural stem cells were generated from the cortex of E14 mice and characterized by immunocytochemistry. A small proportion of the primary cells generated neurospheres after 7 days of initial culture (Fig. 1a). When NSCs were cultured on poly-l-ornithine hydrobromide and laminin-coated plates, they were able to grow as monolayers with adherence to the plate (Fig. 1b). Immunostaining analysis showed that cells were Nestin^+^ and Sox2^+^ while GFAP^–^ and DCX^–^ (Fig. 1c–f), suggesting that the majority of the cells in the culture maintained a stem cell phenotype.

### Differentiation and proliferation of NSCs after pretreatment with adjudin

In order to explore whether adjudin could affect the differentiation and proliferation of NSCs, cells were cultured in a monolayer and pretreated with adjudin at a concentration of 10 or 30 μM. The results of immunostaining indicated that NSCs under the two concentrations of adjudin pretreatment were positive for Nestin and Sox2, and negative for DCX, whereas GFAP was negative for 10 μM and positive for 30 μM pretreated NSCs (Additional file 1: Figure S1a). Fluorescent photomicrographs of Ki67 showed that 10 μM adjudin did not affect the proliferation of NSCs, but 30 μM adjudin could apparently inhibit NSC proliferation (Additional file 1: Figure S1b). Both results suggest that 10 μM adjudin had no effect on the differentiation and proliferation of NSCs.

### Adjudin preconditioning improved the survival of and maintained the ATP level of NSCs under H_2_O_2_ stress

To evaluate whether adjudin preconditioning could reduce NSC death under stress in vitro, we used hydrogen peroxide oxidative stress models. We first investigated the effect of different concentrations of adjudin and H_2_O_2_ on the cell viability of NSCs in order to establish the working concentration of adjudin and H_2_O_2_. After pretreatment of adjudin for 24 hours, the LDH assay revealed that adjudin could not induce cell death even at 60 μM (Additional file 2: Figure S2a), but the results of the CCK-8 assay showed that 30 and 60 μM adjudin could significantly decrease cell viability, while 5 and 10 μM adjudin had no effect on this (Additional file 2: Figure S2b). Combining with the immunostaining results of Ki67 (Additional file 1: Figure S1b), we inferred that this was because high concentrations of adjudin could inhibit cell proliferation instead of decreasing NSC viability. As shown in Additional file 2: Figure S2b, treatment with H_2_O_2_ reduced NSC viability significantly in a concentration-dependent manner. The optimal concentration of H_2_O_2_ for subsequent experiments was determined to be 0.1 mM H_2_O_2_ because cell viability was 40–50% at this concentration (Additional file 2: Figure S2c, d).

**Fig. 2:**
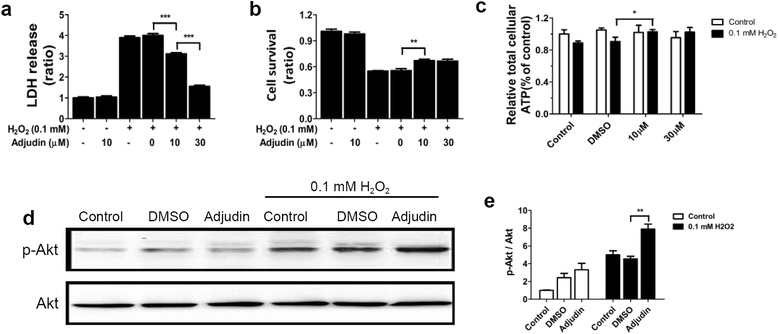
Adjudin pretreatment attenuated cell death and maintained the ATP level of NSCs after H_2_O_2_ stimulation. Assays to evaluate whether adjudin pretreatment could attenuate cell death of NSCs after 0.1 mM H_2_O_2_ exposure. Cell death and cell survival measured by LDH (**a**) and CCK-8 assay (**b**). ATP level of NSCs after H_2_O_2_ stimulation with/without adjudin pretreatment (**c**). Levels of p-Akt and Akt detected by western blot analysis after H_2_O_2_ treatment (**d**). Quantification of the densitometric value of protein bands normalized to total Akt (**e**). Bars represent mean ± SEM from three independent experiments. *P < 0.05, **P < 0.01, ***P < 0.001. LDH lactate dehydrogenase

After 1 hour of 0.1 mM H_2_O_2_ stimulation, cells were replenished with fresh medium and cultured for another 24 hours, to be followed with the LDH assay and CCK-8 assay, which revealed that adjudin-preconditioned NSCs (10 and 30 μM) had a significant reduction in death and an increase in cell survival, compared with the nonpreconditioned NSCs (Fig. 2a, b). This cytoprotective effect was supported by the ATP assay as adjudin pretreatment could maintain the ATP level of NSCs after H_2_O_2_ stimulation (Fig. 2c). The serine/threonine kinase Akt, which is a conserved family of signal transduction enzymes, not only plays a pivotal role in the cell death/survival pathway [40, 41] but also plays an important part in regulating inflammatory responses and apoptosis [42]. Here we used western blot analysis to estimate the activity of the Akt signaling. Compared to the nonpreconditioned NSCs, adjudin pretreatment could dramatically increase the ratio of p-Akt/Akt after H_2_O_2_ stimulation (Fig. 2d, e).

### Adjudin preconditioning upregulated antioxidant genes and reduced oxidative stress in vitro

**Fig. 3:**
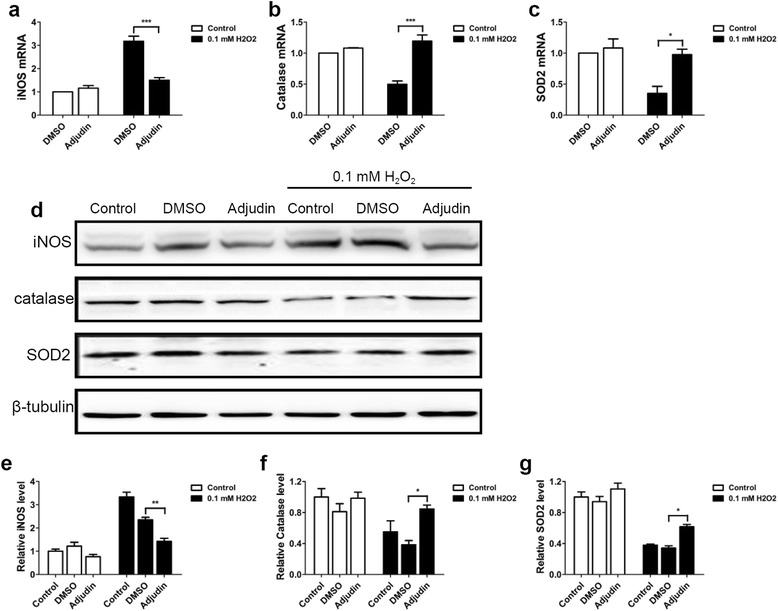
Adjudin-pretreated NSCs inhibited H_2_O_2_-induced oxidative stress. Bar graphs show mRNA levels of iNOS, catalase, and SOD2: relative mRNA expression of iNOS (**a**), catalase (**b**), and SOD2 (**c**) normalized to Rplp0. Adjudin-pretreated NSCs inhibited H_2_O_2_-induced oxidative stress at protein levels. Representative western blot analysis showed that adjudin inhibited H_2_O_2_-induced iNOS upregulation, and increased catalase and SOD2 protein levels in the presence of 0.1 mM H_2_O_2_ (**d**). Quantification of densitometric value of the protein bands normalized to the respective β-tubulin (**e**–**g**). Bars represent mean ± SEM from three independent experiments. *P < 0.05, **P < 0.01, ***P < 0.001

We next sought to elucidate the underlying mechanism of adjudin-induced cytoprotection. As exogenous H_2_O_2_ could induce a strong increase in intracellular ROS levels within 1 hour of cell treatment [43], we investigated the expression of iNOS and several antioxidant genes using RT-PCR and western blot analysis. Real-time RT-PCR assays showed that adjudin preconditioning significantly inhibited iNOS level (Fig. 3a) and upregulated expression of catalase (Fig. 3b), SOD2 (Fig. 3c), and GCLC (Additional file 3: Figure S3a) after 1 hour of H_2_O_2_ stimulation followed by 12 hours of reculture, whereas it did not change NOX4, HO-1, NQO1, and Nrf2 levels (Additional file 3: Figure S3b–e). This was also supported by western blot analysis of the whole cell lysate from the NSCs, showing that adjudin significantly lowered protein expression of iNOS and induced higher levels of catalase and SOD2 after 1 hour of H_2_O_2_ stimulation followed by 24 hours of normal condition culture (Fig. 3d–g). This finding suggested that resistance to oxidative stress is one of the mechanisms of adjudin-induced cytoprotection.

### Adjudin preconditioning promoted expression of neurotrophic factors in vitro

**Fig. 4:**
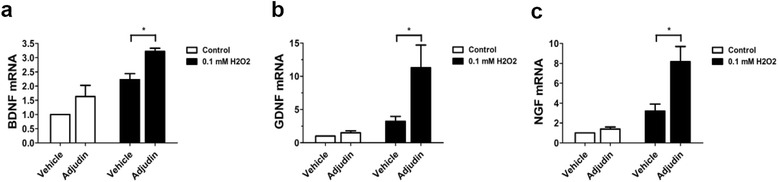
Induction of neurotrophic factors with adjudin preconditioning in vitro. Real-time RT-PCR assays of NSCs. Relative mRNA expressions of BDNF, NGF, and GDNF normalized to Rplp0 (**a**–**c**). Bars represent mean ± SEM from three independent experiments. *P < 0.05. BDNF brain-derived neurotrophic factor, GDNF glial cell-derived neurotrophic factor, NGF nerve growth factor

Because NSCs could secrete many neurotrophic factors and other soluble molecules to modify the release of inflammatory mediators and oxidative reaction [13, 27, 44], we tested whether adjudin changed their expression in NSCs in vitro. Significantly higher gene expression of BDNF, nerve growth factor (NGF), and glial cell-derived neurotrophic factor (GDNF) was detected in the adjudin-preconditioned NSC group after 1 hour of H_2_O_2_ stimulation and 12 hours of reculture, compared with the nonpreconditioning NSC group (Fig. 4a–c).

### Adjudin preconditioning reduced brain infarct volume and improved neurobehavioral outcome after ischemia/reperfusion

**Fig. 5:**
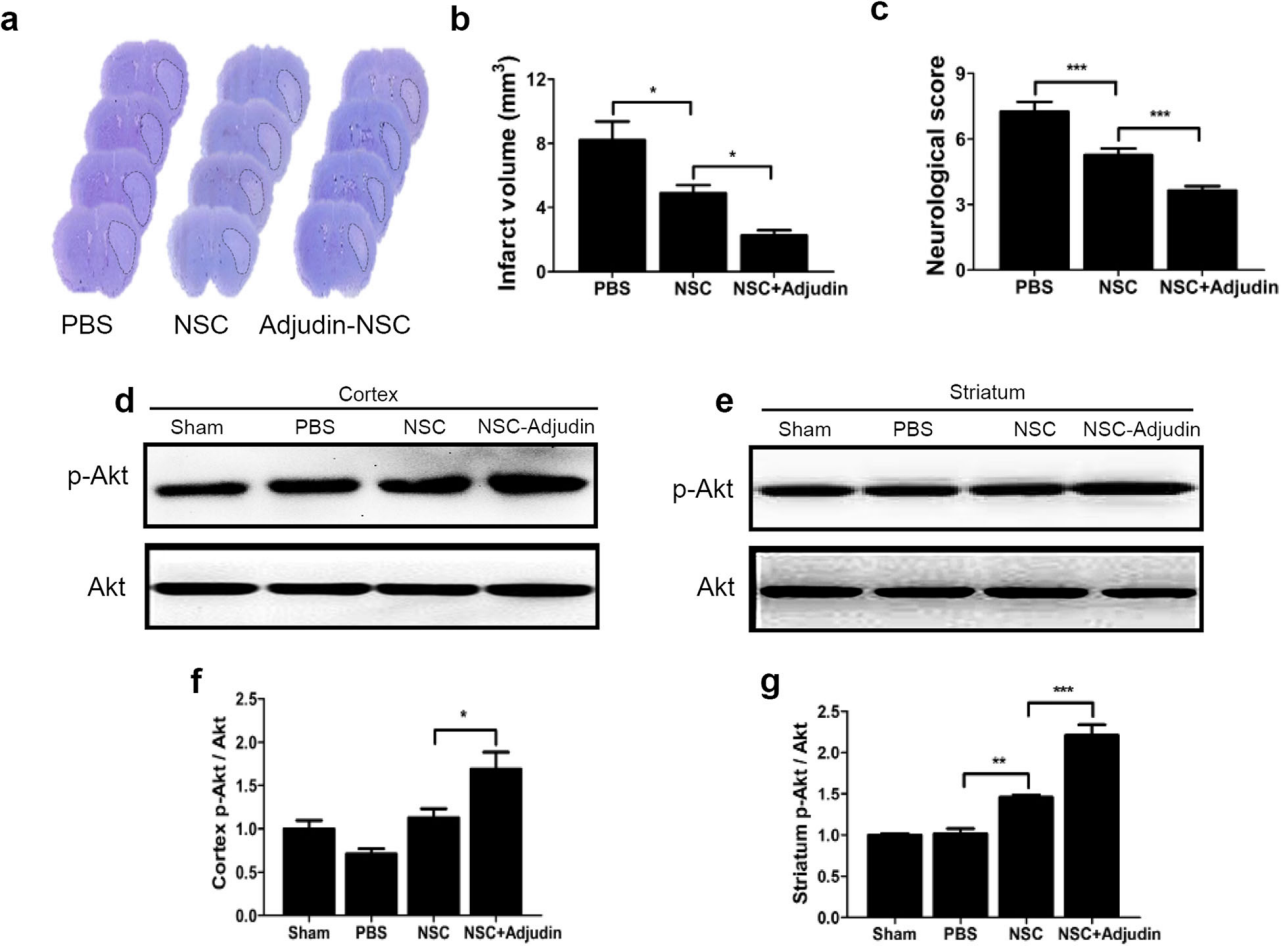
Adjudin-pretreated NSCs reduced brain infarct volume and improved neurobehavioral outcome after I/R. Representative sets of cresyl violet staining of brain sections from mice treated with PBS, untreated NSCs, and adjudin-pretreated NSCs 3 days following tMCAO. Dashed line shows the border of the infarct area (**a**). Quantification of infarct volumes (**b**). *n* = 8 in each group. Adjudin-pretreated NSCs significantly ameliorated neurological deficits 3 days after transplantation when compared to the PBS or NSC group. *n* = 14 for PBS and untreated NSC group, and *n* = 19 for adjudin-pretreated NSC group (**c**). Adjudin-pretreated NSCs promoted the phosphorylation of Akt in ipsilateral cortex (**d**) and striatum (**e**) after tMCAO. Representative western blot assay showing that adjudin increased the p-Akt protein level 3 days after tMCAO compared with sham, PBS, and NSC groups. Quantification of densitometric value of the protein bands of cortex and straitum normalized to total Akt (**f**, **g**). *n* = 6 in each group. Data are mean ± SEM. *P < 0.05, **P < 0.01, ***P < 0.001. NSC neural stem cell, PBS phosphate-buffered saline

Twenty-four hours after tMCAO, mice were divided randomly into three groups for NSC or vehicle injection: PBS group, NSC group, and adjudin-pretreated NSC group. NSCs (1 × 10^6^ cells suspended in PBS) that was pretreated with or without adjudin or untreated were injected into the striatum of the ipsilateral hemisphere in mice. Brain infarct volume was determined by cresyl violet staining 3 days after cell transplantation (Fig. 5a). Adjudin-pretreated NSCs greatly reduced infarct volume by as much as 50% compared to the PBS group, whereas untreated NSCs only produced ~ 30% reduction in the infarct volume compared to the PBS group (Fig. 5b). Meanwhile, adjudin preconditioning improved behavioral performance with the neuroscore plummeting by approximately 50% in comparison to the PBS group, while untreated NSCs resulted in only a 25% decrease in neuroscore (Fig. 5c). These findings illustrate that adjudin pretreatment could significantly attenuate I/R-induced cerebral injury. Moreover, compared to the untreated NSCs, PBS, and sham groups, the adjudin preconditioning group considerably increased the ratio of p-Akt/Akt both in the cortex and the striatum (Fig. 5d–g).

### Adjudin preconditioning reduced cytokine production and attenuated microglial activation after ischemia/reperfusion

**Fig. 6:**
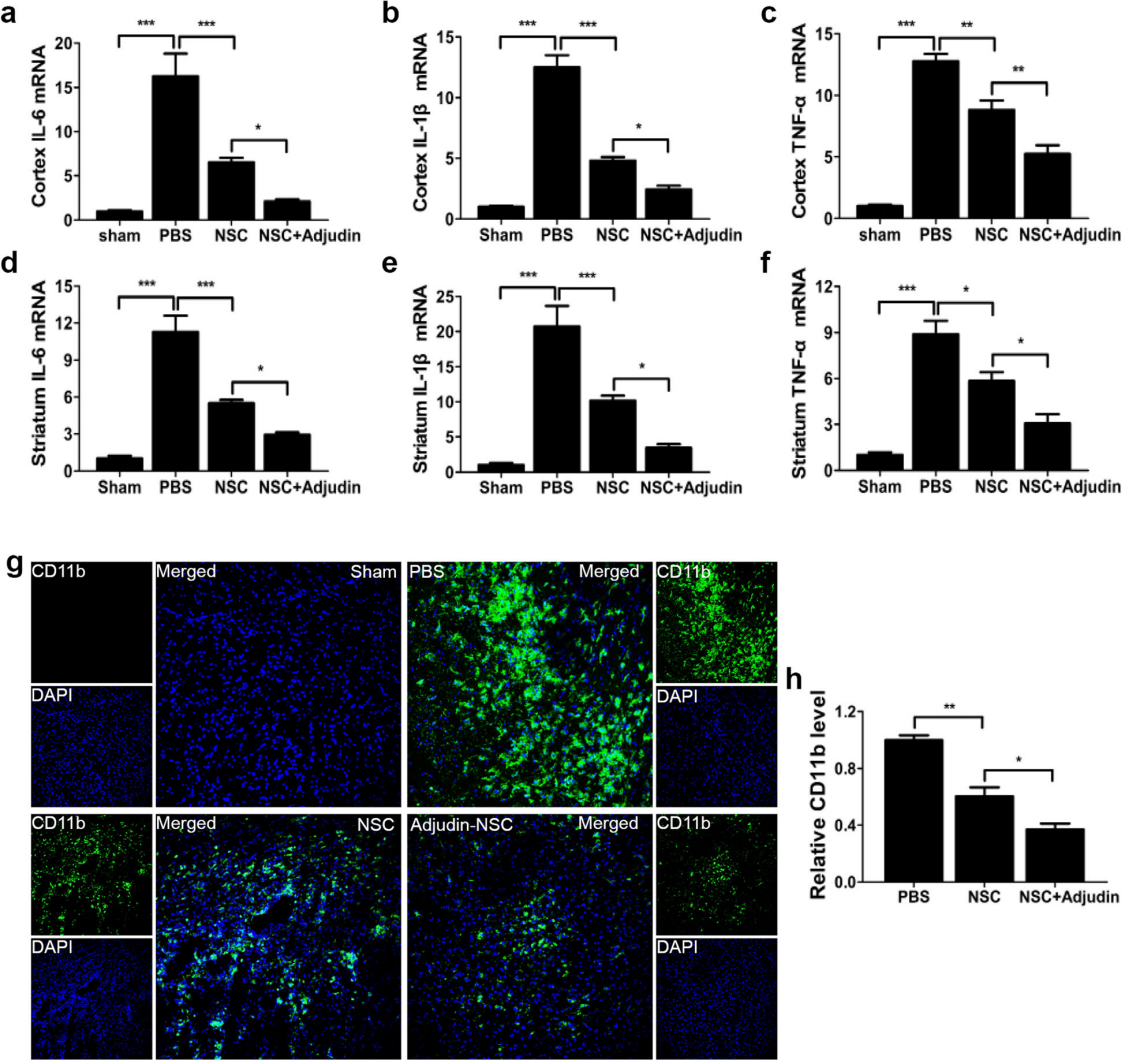
Adjudin-pretreated NSCs inhibited cytokine production and activation of microglia after I/R. Relative mRNA expression of IL-6, IL-1β, and TNF-α normalized to Rplp0 detected 3 days following cell transplantation. Expression of IL-6 (**a**, **d**), IL-1β (**b**, **e**), and TNF-α (**c**, **f**) in ipsilateral cortex and striatum shown in the NSC and adjudin-pretreated NSC groups. *n* =6 in each group. Immunofluorescence staining for CD11b (green) in the sham group, and tMCAO groups with either PBS injection, NSC injection, or adjudin-pretreated NSC injection. Samples were acquired 3 days after cell transplantation, with DAPI staining for contrast (**g**). Scale bar =100 μm. Quantification of CD11b immunofluorescence intensity in each group (**h**). *n* = 8 in each group. Data are mean ± SEM. *P < 0.05, **P < 0.01, ***P < 0.001. DAPI 4,6-diamidino-2-phenylindole, NSC neural stem cell, PBS phosphate-buffered saline

To investigate whether adjudin-pretreated NSCs in the acute phase of cerebral ischemia had a better effect on immunomodulatory influence, we first examined IL-6, IL-1β, and TNF-α mRNA expression in both the cortex and the striatum. The results showed that IL-6, IL-1β, and TNF-α mRNA were increased dramatically at day 3 following tMCAO. The expression of the three cytokines decreased significantly in the untreated NSC group compared to the PBS group, and in the adjudin-pretreated NSC group, further reduction in their expression was found (Fig. 6a–f). As the resident immune cells in the central nervous system (CNS), microglia could be activated by I/R injury, which could regulate the primary events of neuroinflammatory responses [45]. We then investigated whether adjudin preconditioning also affected microglia in the tMCAO model. A CD11b signal, an indicator of active microglia, was revealed by fluorescence microscopy (Fig. 6g, h). In the sham group, no obvious activation of microglia and CD11b signal were detected (Fig. 6g top left panel). In the PBS group, strong staining of CD11b was widely found in the ipsilateral hemisphere (Fig. 6g top right panel). Contrarily, stereotactic injection of nonpreconditioned NSCs after reperfusion significantly inhibited the activation of microglia (Fig. 6g bottom left panel). Moreover, adjudin-pretreated NSCs could further inhibit microglia activation, where much less CD11b signal was detected (Fig. 6g bottom right panel). Statistical analysis of the CD11b signal from brain sections of mice indicated that adjudin preconditioning significantly attenuated microglial activation in the ipsilateral region of the brain after I/R injury (Fig. 6h).

### Adjudin preconditioning suppressed M1 microglia activation but promoted M2 polarization after ischemia/reperfusion

As the principal immune cells in the brain, microglia cells share certain characteristics with macrophages and response to immunoreaction for local CNS injury [7]. M1 macrophage polarization can produce proinflammatory cytokines, such as IL-6, IL-1β, and TNF-α, and express markers such as CD16 [46]. Activated M2 polarized microglia can express arginase-1 (Arg-1), CD163, Ym1, produce other anti-inflammation cytokines and restore homeostasis [20, 24, 47].

**Fig. 7:**
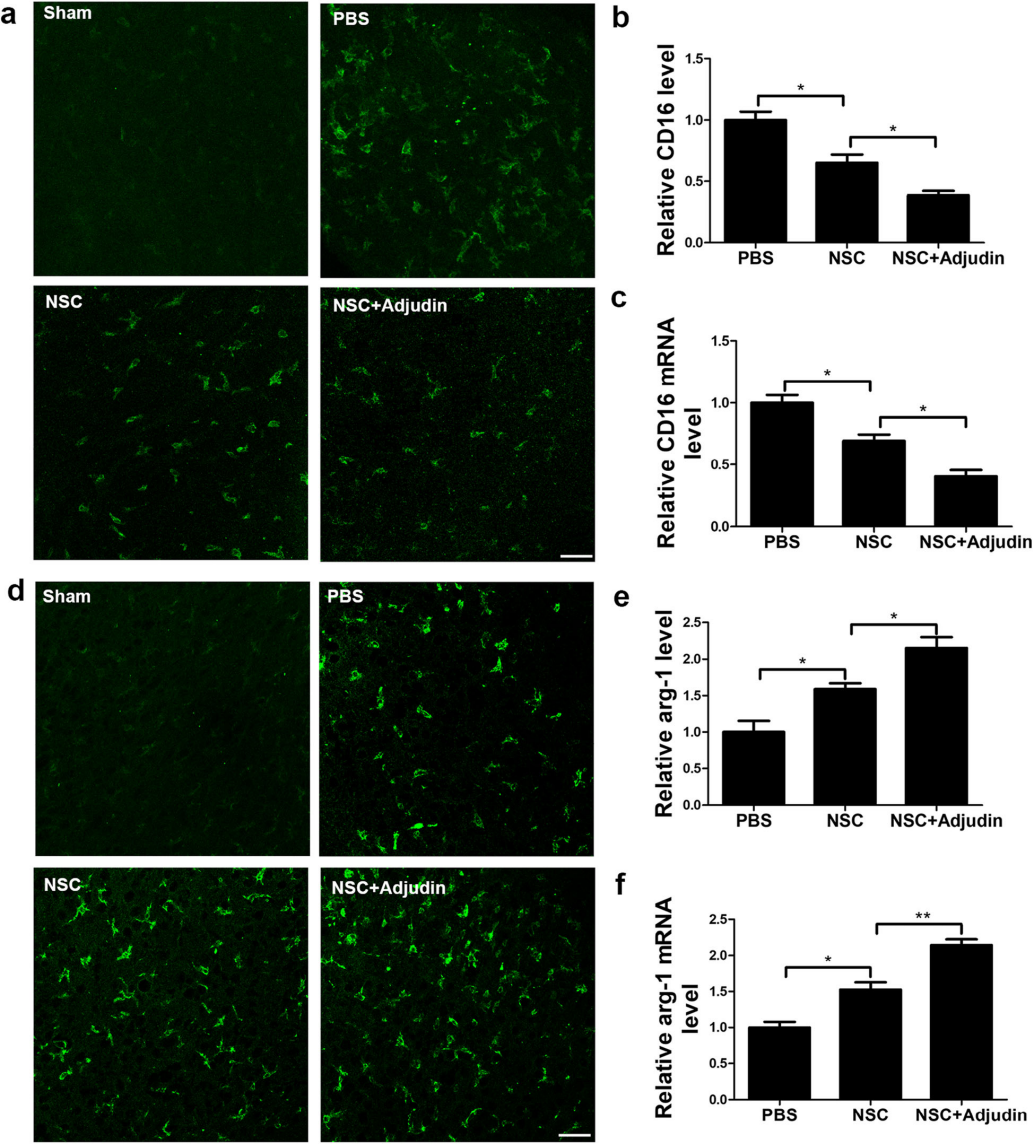
Adjudin-pretreated NSCs inhibited M1 activation but promoted M2 expression after I/R. Immunofluorescence staining for CD16 and Arg-1 showed activation of M1 and M2 microphages in sham, PBS, nonpretreated NSC, and adjudin-pretreated NSC groups at 3 days after cell transplantation (**a**, **d**). Scale bar =50 μm. Quantification of MI/M2 positive cells in each group (**b**, **e**). Relative mRNA expression level of CD16 and Arg-1 checked using RT-PCR normalized to Rplp0 (**c**, **f**). *n* = 5 in each group. Data are mean ± SEM. *P < 0.05, **P < 0.01. NSC neural stem cell, PBS phosphate-buffered saline

It has been reported that the dynamic changes of M1/M2 macrophage activation are involved in CNS damage and regeneration. M1/M2 macrophage polarization also plays an important role in controlling the balance between promoting and suppressing inflammation [20, 48]. Here we stained CD16 and Arg-1 to check MI/M2 microglial activation. The results revealed that ischemic brain damage could prominently activate both M1 and M2 microglia compared with sham groups (Fig. 7a, d). Furthermore, when comparing adjudin-pretreated NSC groups with nonpretreated NSC groups, we found that adjudin pretreatment could significantly suppress the expression of M1 microglia and promote M2 microglia expression (Fig. 7a–f).

### Adjudin preconditioning attenuated oxidative stress after ischemia/reperfusion

**Fig. 8:**
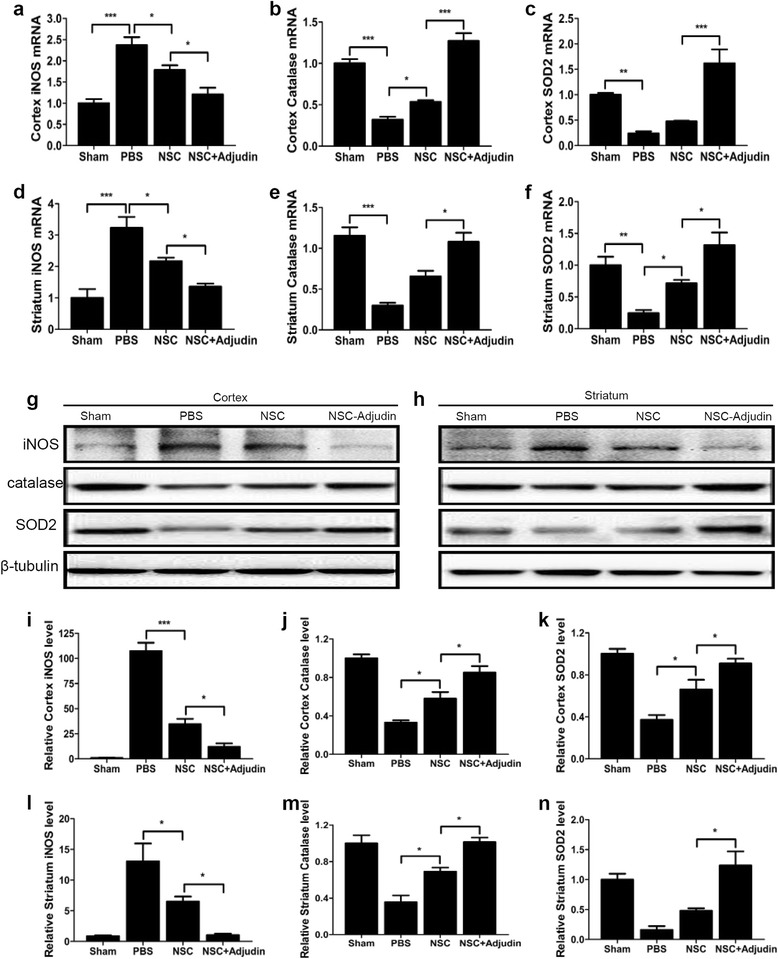
Adjudin-pretreated NSCs inhibited the oxidative stress after tMCAO. Modulation of oxidative stress gene expression in vivo. Relative mRNA expression of iNOS, catalase, and SOD2 in ipsilateral cortex (**a**–**c**) and striatum (**d**–**f**) normalized to Rplp0 detected 3 days after cell transplantation. Western blot analysis of iNOS, catalase, and SOD2 protein levels in ipsilateral cortex and striatum 3 days after cell transplantation (**g**, **h**). Quantification of densitometric value of the protein bands of cortex (**i**–**k**) and striatum (**l**–**n**) normalized to the respective β-tubulin. *n* = 6 in each group. Data are mean ± SEM. *P < 0.05, **P < 0. 01, ***P < 0. 001. NSC neural stem cell, PBS phosphate-buffered saline

Since ROS also plays an important role in cerebral I/R injury, we then investigated the effect of adjudin preconditioning on resistance to oxidative stress. Compared with the PBS and NSC groups, the iNOS mRNA level was significantly decreased in the adjudin preconditioning group both in the cortex and striatum (Fig. 8a, d). Also, the expression of antioxidant genes catalase (Fig. 8b, e) and SOD2 (Fig. 8c, f) was apparently increased in the adjudin pretreatment group after I/R injury. Western blot analysis of whole cell lysate from the ipsilateral cortex and striatum also supported these results, which showed that adjudin preconditioning dramatically decreased iNOS protein expression and promoted higher levels of catalase and SOD2 3 days after I/R (Fig. 8g–n).

### Adjudin preconditioning enhanced neuroprotection after tMCAO via p-38 and JNK but not the ERK signaling pathway

**Fig. 9:**
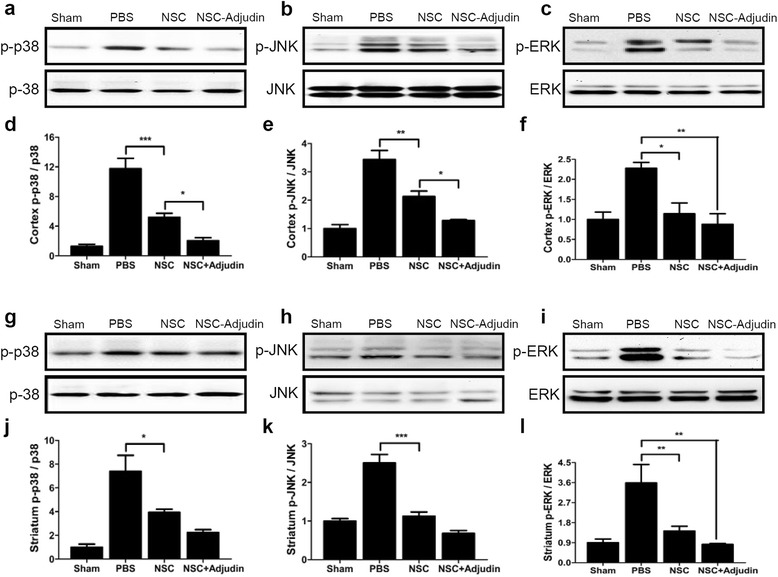
Adjudin-pretreated NSCs inhibited phosphorylation of p-38 and JNK after tMCAO. P-p38, p-38, p-JNK, JNK, p-ERK, and ERK levels in sham, PBS, nonpretreated NSC, and adjudin-pretreated NSC groups at 3 days after cell transplantation in the ipsilateral cortex (**a**–**f**) and striatum (**g**–**l**). Quantification of densitometric values of the protein bands normalized to total p38, JNK, and ERK1/2 (**d**–**f**, **j**–**l**). *n* = 6 in each group. Data are mean ± SEM. *P < 0.05, ***P < 0.001. NSC neural stem cell, PBS phosphate-buffered saline

To assess the phosphorylation status of the MAPK signaling pathway, western blot analysis was used. We demonstrated that I/R significantly increased p38, JNK, and ERK1/2 phosphorylation levels in the cortex and striatum compared with sham, and this induction could be inhibited after transplantation of NSCs (Fig. 9a, c). However, compared with the nonpretreated NSC group, the adjudin preconditioning group had a more profound effect on inhibiting the phosphorylation level of p38 and JNK in the cortex (Fig. 9a, b, d, e), while ERK1/2 (Fig. 9c, f) phosphorylation had no detectable changes after transplantation between groups. No significant differences were observed in the expression of total ERK1/2, total JNK1/2, and total p38 MAPKs among all experimental groups. Therefore, the results indicated that I/R induced inflammatory cytokines and oxidative stress by activating the p38 and JNK pathway but not the ERK signaling pathway.

### Adjudin preconditioning attenuated ischemia/reperfusion-induced blood–brain barrier leakage

**Fig. 10:**
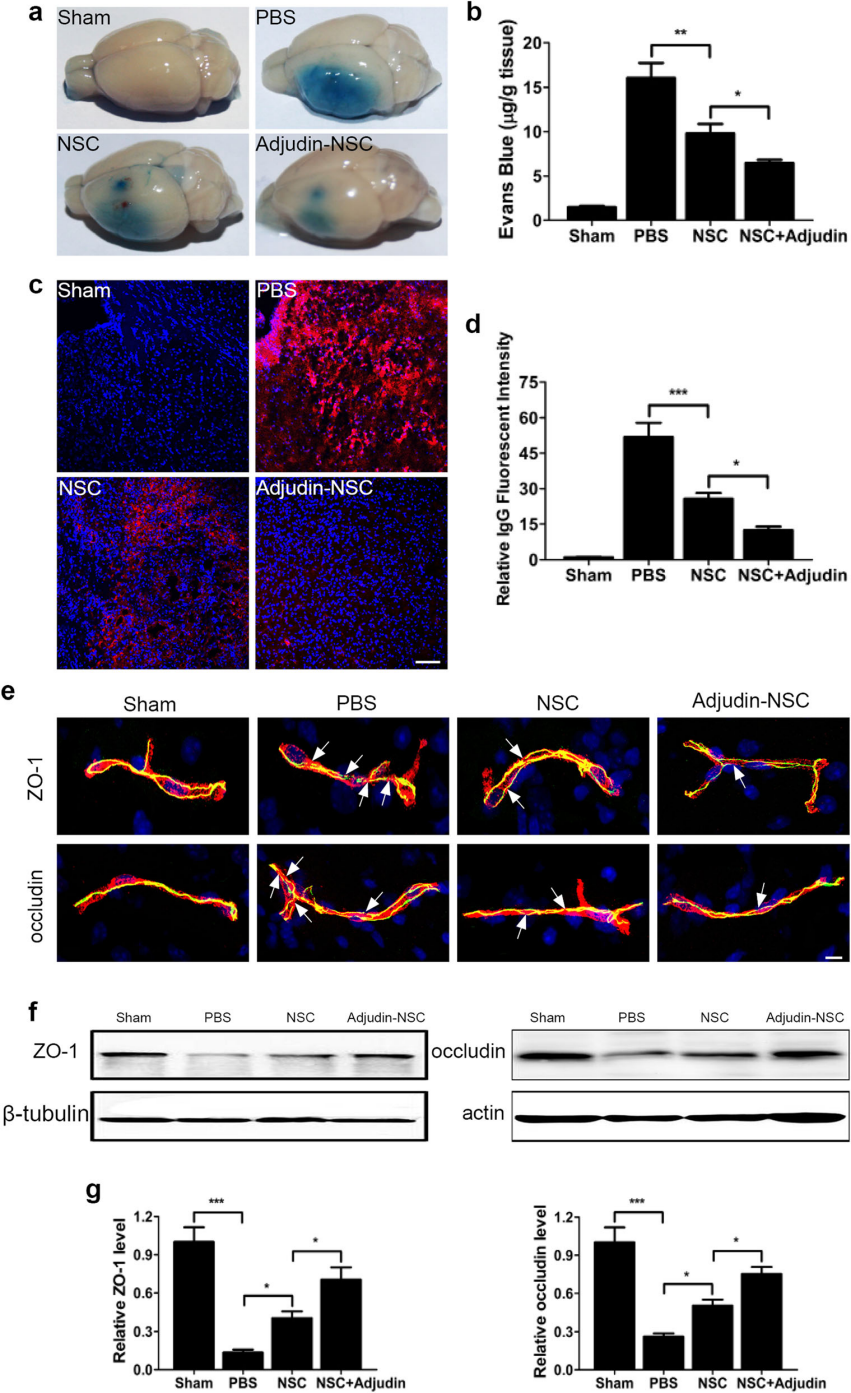
Adjudin-pretreated NSCs lessened Evans blue and IgG extravasation and inhibited ZO-1 and occludin degradation. Photographs represent the perfused brains after EB injection (**a**). Quantification of extravasated EB dye. The dye was analyzed by a spectrophotometer at 610 nm (**b**). *n* =14 for PBS and untreated NSC groups, and *n* = 19 for adjudin-pretreated NSC group. Immunofluorescence staining for IgG (red) in sham, PBS, nonpretreated NSC, and adjudin-pretreated NSC groups at 3 days after cell transplantation, with DAPI staining for contrast (**c**). Scale bar =100 μm. Quantification of the IgG fluorescent intensity in each group (**d**). *n* = 8 in each group. Sections from ischemic penumbra were stained for ZO-1 (green) and occludin (green), and then costained with endothelial marker CD31 (red) (**e**). Nuclei were stained with DAPI. Scale bar = 100 μm. Representative western blot analysis for ZO-1 and occludin protein levels in the ischemic penumbra from sham, PBS, nonpretreated NSC, and adjudin-pretreated NSC groups at 3 days after cell transplantation (**f**). Quantification of densitometric values of the protein bands normalized to the respective β-tubulin and actin (**g**). *n* = 6 in each group. Data are mean ± SEM. *P < 0.05, **P < 0.01, ***P < 0.001. NSC neural stem cell, PBS phosphate-buffered saline

The permeability of BBB after ischemic brain injury was assessed by measuring the extravasation of EB and IgG protein, which could not leak to the brain parenchyma through the BBB in the normal physiological state. We demonstrated that a tremendous amount of EB and IgG were detected in the ipsilateral hemisphere of the PBS group, while NSCs remarkably reduced the EB and IgG leakage, and adjudin-pretreated NSCs could further decrease the leakage of EB and IgG, which indicated that BBB integrity was even better protected by adjudin-pretreated NSCs (Fig. 10a–d). Meanwhile, we also found in the sham group that no EB dye or IgG signal was detected in the same brain regions (Fig. 10a–d). To investigate the mechanism of BBB disruption, we analyzed the localization of tight junction (TJ)-related proteins ZO-1 and occludin in cerebral vascular structures by immunofluorescence microscopy in conjunction with CD31, an endothelial marker that also locates at the BBB, and by western blot analysis to determine the change of the protein levels. Confocal microscopy analysis showed that ZO-1 and occludin positive staining were continuously located on the endothelial cell margin of cerebral microvessels in the sham group, while this continuity was disrupted after I/R injury by forming many gaps along the microvessels (Fig. 10e). However, this process could be reversed by stereotactic injection of NSCs, and compared with the nonpretreated NSC group, adjudin preconditioning could further lessen gap formation after tMCAO (Fig. 10e). To corroborate this result, western blot analysis of lysates from the ipsilateral region was adopted. We found that the significant reduction of ZO-1 and occludin levels after I/R (PBS versus sham) could be rescued by NSC transplantation and adjudin preconditioning had a better effect on protecting against the protein reduction of ZO-1 and occludin after I/R injury (Fig. 10f). Together, these results further demonstrated that the BBB destruction after I/R injury could be effectively rescued by adjudin-pretreated NSCs.

### Adjudin preconditioning enhanced the secretion of neurotrophic factors after ischemia/reperfusion

**Fig. 11:**
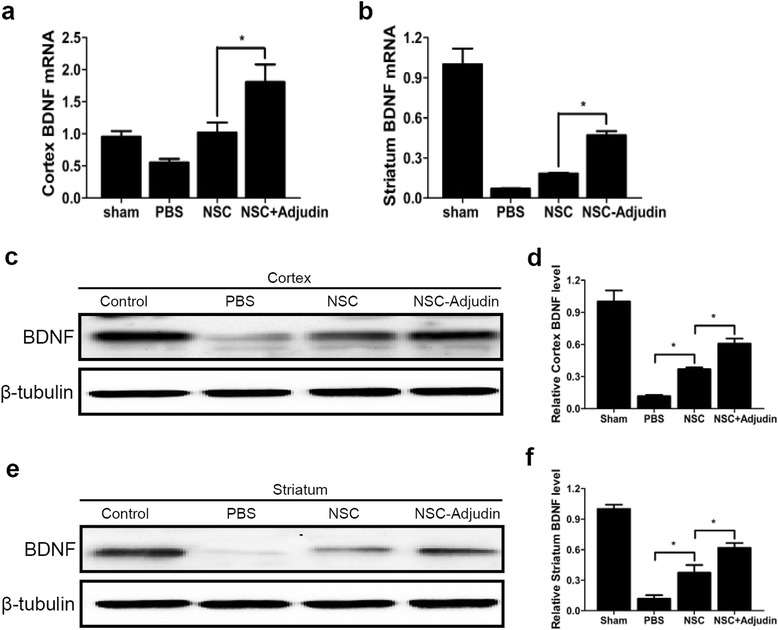
Adjudin-pretreated NSCs upregulated expression of neurotrophic factors. Relative mRNA expression of BDNF, NGF, and GDNF normalized to Rplp0 in cortex (**a**) and striatum (**b**) from sham, PBS, nonpretreated NSC, and adjudin-pretreated NSC groups at 3 days after cell transplantation. Western blot analysis of BDNF in cortex (**c**, **d**) and striatum (**e**, **f**) from sham, PBS, nonpretreated NSC, and adjudin-pretreated NSC groups at 3 days after cell transplantation. Quantification of densitometric values of the protein bands normalized to β-tubulin. *n* = 6 in each group. Data are mean ± SEM. *P < 0.05, ***P < 0.001. BDNF brain-derived neurotrophic factor, NSC neural stem cell, PBS phosphate-buffered saline

To evaluate the ability of NSCs to secrete neurotrophic factors, we measured BDNF levels in both the cortex and striatum of the ipsilateral hemisphere using RT-PCR and western blot analysis 3 days after ischemia and transplantation. Real-time RT-PCR assays showed that these paracrine factors significantly increased in the adjudin-pretreated NSC group compared with the nonpretreated NSC group and the PBS group (Fig. 11a, b), which were also confirmed by western blot analysis (Fig. 11c–f).

### Adjudin preconditioning promoted angiogenesis and enhanced neurobehavioral recovery after ischemia/reperfusion

**Fig. 12:**
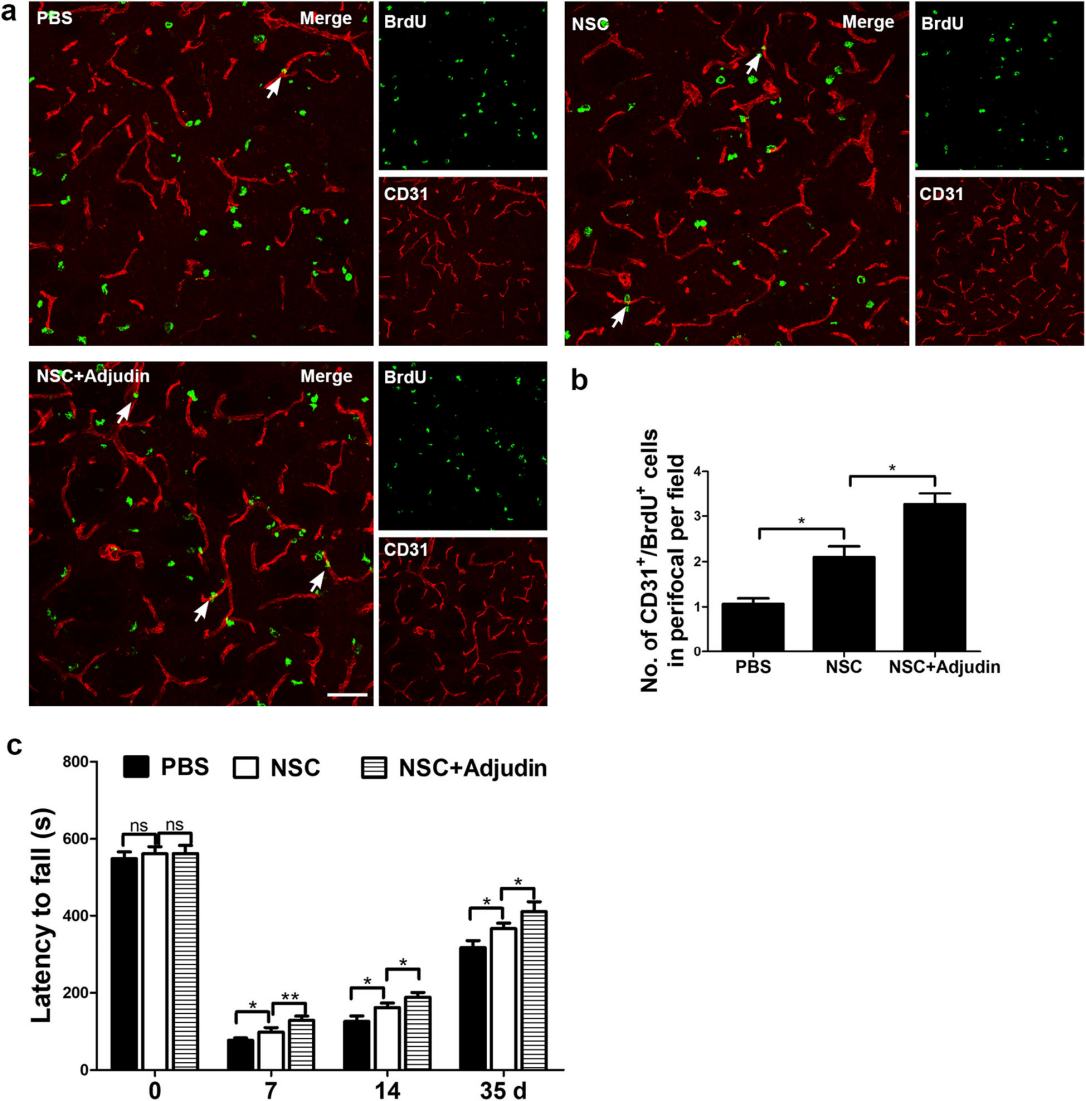
Immunostaining for CD31 (red) and BrdU (green) showing the number of new generated vessels from PBS, nonpretreated NSC, and adjudin-pretreated NSC groups at 35 days after cell transplantation (**a**). Scale bar =50 μm. Quantification of CD31^+^/BrdU^+^ cell number in each group (**b**). *n* = 5 in each group. Bar graphs summarizing results of rotarod test maintaining time in each group (**c**). *n* = 5 in each group. Data are mean ± SEM. *P < 0.05, **P < 0.01. NSC neural stem cell, PBS phosphate-buffered saline

Ischemic angiogenesis directly relates to reestablishment of microcirculation within the I/R damaged area and represents a key vital process for poststroke functional recovery [49, 50]. Since angiogenesis could modulate the endogenous angiogenic response to generate new vessels and then increase blood supply which is necessary for new neuronal survival and development, angiogenesis is directly linked to neurogenesis [51, 52]. Here we further measured EPC marker CD31 and 5-bromo-2′-deoxyuridine (BrdU) double-positive cells to evaluate angiogenesis 35 days after transplantation (Fig. 12a, b). The staining results showed that nonpreconditioned NSCs could significantly increase new vessel generation compared with the PBS group (Fig. 12a top panel, b), while the adjudin-pretreated NSCs had a more remarkable effect on angiogenesis (Fig. 12a bottom left panel, b). To evaluate the effect of adjudin pretreatment on functional recovery, rotarod test was performed at different time points (≤5 weeks) after cell transplantation.

The rotarod maintaining time declined sharply after tMCAO surgery compared with the nonoperative group (Fig. 12c), while the maintaining time was significantly prolonged in surgery groups 7, 14, and 35 days after cell transplantation (Fig. 12c). In each tMCAO group, we found that the functional recovery effects were in accordance with angiogenesis results. NSC transplantation could significantly increase the rotarod maintaining time compared with the PBS group, and adjudin-pretreated NSCs showed even better effects (Fig. 12c).

## Discussion

In this study, we showed that, compared with nonpreconditioned NSCs, adjudin preconditioning not only enhanced the survival rate of NSCs under H_2_O_2_ oxidative stress in vitro, but also had a better effect on decreasing infarct volume, improving behavioral outcome, inhibiting neuroinflammation and oxidative stress, maintaining BBB integrity, and expressing higher levels of neurotrophic factors, resulting in stronger therapeutic effects in I/R-induced brain injury. Such a neuroprotective effect was mediated by inhibiting activation of the p38 and JNK MAPK signaling pathway. Together our results suggested the potential of using adjudin for NSC transplantation, and provided preclinical experimental evidence for the combination therapy of adjudin and NSCs after stroke.

Because of the complexity of the ischemic cascade, which includes various mechanisms of excitotoxicity (glutamate release and receptor activation), calcium influx, ROS scavenging, NO production, inflammatory reactions, and apoptosis, numerous molecular targets have been tackled in order to achieve neuroprotection [53, 54]. Since the majority of patients continue to exhibit neurological deficits even following successful thrombolysis and therapy, restorative therapies are urgently needed to promote brain remodeling and repair once stroke injury has occurred. Stem cell transplantation therapy has emerged as a promising regenerative medicine for ischemic stroke which could promote tissue repair and functional recovery via potent immune modulatory actions, trophic support enforcement, and cell replacement mechanisms [13, 55]. However, a number of issues and problems remain unresolved and need specific attention in order to develop clinical treatments successfully. These include an appropriate cell source in consideration of therapeutic value and ethical concerns, cell type-specific differentiation, and survival of transplanted cells in the harsh pathological microenvironment [16]. Massive death of donor cells in the infarcted area during acute phase immensely lowers the efficacy of the procedure [17]. In order to improve the effect of stem cell-based therapy, various strategies are discussed which have been adopted to develop and optimize the protocols to enhance donor stem cell survival post transplantation, with special focus on the preconditioning approach [56]. Up to now, a number of preconditioning triggers have been tested in stem cell-based therapy, such as ischemia, hypoxia, H_2_O_2_, erythropoietin (EPO), insulin-like growth factor-1 (IGF-1), pharmacological agents, and so forth, which have shown that exposure of stem cells to sublethal hypoxia or other preconditioning insults increased the tolerance of these cells to multiple injurious insults and thus protected them against the harsh environment after transplantation [27, 57–61].

Many studies have already illustrated that NSC therapy has great potential to restore neurological function after ischemic brain injury [6, 14], and here we likewise demonstrated the neuroprotection effect of NSCs which attenuated infract volume and improved the outcome of behavioral recovery after stroke and transplantation onward. In our study, we found that the MAPK signaling pathway as one of the underlying mechanisms of stem cell function, was dramatically inhibited 3 days after NSC transplantation. Our results showed that NSC transplantation could inhibit the activation of p-ERK1/2, p-JNK1/2, and p-p38 MAPKs which could significantly increase after I/R injury in comparison with that of sham-operated animals. MAPK signaling pathways are not only implicated in inflammatory and apoptotic processes of cerebral I/R injury, but are also involved in the proliferation, survival, and cell fate determination (neurogenesis vs gliogenesis) of NSCs that depend on the extrinsic factors regulated by different MAPK-activated transcription factors, or interacted with other signaling pathways [62, 63]. MAPKs are activated after focal cerebral I/R, which mainly function as mediators of cellular stress by phosphorylating intracellular enzymes, transcription factors, and cytosolic proteins involved in cell survival, inflammatory mediator production, and apoptosis [64, 65]. Kyriakis and Avruch showed that the presence of JNK and p38 MAPKs had an effect on cell injury, unlike the ERK signaling that was part of the survival route [64]. Cumulative experimental evidence showed that p38 and JNK MAPKs could be activated in neurons, microglia, and astrocytes after various types of ischemia [66–69], and their activation was associated with the production of proinflammatory cytokines, such as TNF-α and IL-1β, which tend to act as perpetrators in the CNS injury [70, 71]. A growing body of evidence showed that inhibition of p38 or JNK MAPK activation using inhibitors or knockout mice could provide protection in a variety of brain injury models [72–75]. However, phosphorylation of ERK occurred at different time intervals after I/R injury. Whether the activation of ERK was associated with neuronal protection or damage in ischemic brain remains to be determined unequivocally [76]. From our experiments, we found that adjudin preconditioning could further decrease the levels of p-JNK1/2 and p-p38 MAPKs, but had no additional effect on the increase of p-ERK1/2 levels compared with that in the nonpreconditioned NSC group. These findings, together with our results, supported the involvement of the JNK1/2 and p38 MAPK pathway in the adjudin preconditioning neuroprotection. Notably, adjudin failing to attenuate the increased p-ERK1/2 levels was consistent with our observations that adjudin treatment did not change p-ERK1/2 levels in H_2_O_2_-induced NSC injury in vitro (Additional file 4: Figure S4).

Adjudin preconditioning could increase the expression of p-Akt both in vitro and in vivo. Akt belongs to a conserved family of signal transduction enzymes, which is the downstream target of phosphoinositide 3-kinase (PI3K) that not only plays an important part in regulating cellular activation and inflammatory responses, but also participates in cell growth, survival, metabolism, and apoptosis [77, 78]. In the initial hours of cerebral ischemia, p-Akt protein level transiently rises in neurons, and this increment is supposed to be a neuroprotective response [79]. The phosphorylation of Akt could activate downstream proteins such as Bcl-2-associated death protein (BAD) and caspase 9, thereby inhibiting the Bax-dependent apoptosis pathway and blocking cytochrome c-mediated caspase 9 activation [78, 80]. In our study, we found that the level of p-Akt was elevated in the adjudin-preconditioned NSC group compared with that of the nonpreconditioned NSC group both in vivo and in vitro. Therefore, we demonstrated that the positive effect of adjudin preconditioning was mediated partially through a PI3K/Akt-dependent mechanism.

Massive cell death is induced in hours to days with additional injury resulting from increased free radicals and inflammatory responses since energy metabolism dysfunction and glutamate excitotoxicity occur in ischemic brain injury [35]. Adjudin preconditioning treatments applied to NSCs have been shown to enhance resistance to those insults by modulating MAPK and Akt signaling pathways, inhibiting the activation of microglia, downregulating IL-6, IL-1β, TNF-α, and iNOS, and upregulating antioxidant genes such as SOD2, catalase, and GCLC. Microglial cells are brain macrophages which serve important functions in many CNS diseases. Our previous work has shown that adjudin could significantly attenuate microglia activation and decrease proinflammatory cytokine release through inhibition of NF-κB activity in BV2 microglia [36], and here we also demonstrated that adjudin-pretreated NSCs could dramatically decrease H_2_O_2_-induced phosphorylation of p65 in NSCs (Additional file 5: Figure S5). Mitochondria play an important role in cytoprotection and preconditioning of cells. Generation of ROS in mitochondria is one of the main triggers that induce ischemic tolerance in the brain [81]. Madhavan et al. [82] demonstrated that NSCs resisted oxidative stress better than neurons because of their higher expression of antioxidant enzymes at a steady state and faster upregulation following oxidative stress stimulation. In this study, we showed that adjudin pretreatment significantly increased SOD2 and catalase activity and decreased iNOS levels in the ischemic penumbra of the cerebral and H_2_O_2_-induced NSC injury compared with nonpreconditioned NSCs. Thus, our results have provided evidence for a better effect of the antioxidative activity of preconditioned NSCs after focal cerebral I/R injury.

Besides neuroinflammation and oxidative stress, we also focused on the protective effects of adjudin-preconditioned NSCs on BBB permeability since maintaining BBB integrity is critical for reducing secondary brain injury following cerebral ischemia. As the core part of the BBB, tight junction proteins like JAM-A, claudin-5, occludin, and ZO-1 are located in the tightly sealed monolayer of brain endothelial cells (BEC) and conferred barrier function to preclude blood substances permeating into the brain parenchyma [35]. Many brain injuries such as ischemia and trauma lead to a disruption and reconstruction of tight junction proteins. In the present study, we demonstrated that, compared with nonpreconditioned NSCs, adjudin preconditioning could further reduce the leakage of IgG and EB by maintaining the protein levels of tight junction protein ZO-1 and occludin, leading to better outcomes in tMCAO mice. This protective effect was due to an attenuation of neuroinflammatory response and oxidative stress, which were capable of disrupting the epithelial barrier by decreasing tight junction protein expression [83].

Better understanding of molecules acting in neuroprotection might illuminate more treatment strategies of neurological disorders [84]. Transplanted NSCs exert beneficial effects not only via structural replacement, but also via neurotrophic actions [85, 86]. An interesting finding of this study was the induction of neurotrophic factors with adjudin preconditioning. Numerous studies have demonstrated that grafted stem cells adapt to the ischemic microenvironment and facilitate homeostasis via the secretion of numerous tissue trophic factors that have beneficial effects on endogenous brain cells, as well as modulatory actions on both innate and adaptive immune responses [13]. Our work illustrated that compared with the nonpreconditioning group, adjudin preconditioning increased the expression of BDNF significantly in the ipsilateral brain 3 days after transplantation. Concomitantly, the heightened expression of BDNF, GDNF, and NGF in vitro in adjudin-pretreated NSCs was consistent with our observations in vivo, further demonstrating the neuroprotection of NSCs preconditioned by adjudin. BDNF withstood cerebral ischemic injury by means of upregulating antioxidant enzymes and mainly interfering with apoptotic pathways [87]. Greenberg et al. [88] found that the Akt pathway was an important downstream signaling pathway of BDNF, and via this pathway BDNF protected tissue from injury and fostered neuronal plasticity. Meanwhile, Lu et al. illustrated that the role of BNDF in hippocampal neurogenesis was mediated by ERK1/2 signaling pathways [89]. Moreover, Almeida et al. revealed that the exposure of neurons to BDNF stimulates CREB phosphorylation and activation via both MAPK and PI3K/Akt pathways. CREB was capable of regulating BDNF gene transcription directly, which suggested that a positive-feedback loop might be operating in some cell populations that were resistant to brain injury [90]. These findings, together with our results, supported that the neuroprotective effects of NSCs and adjudin-preconditioned NSCs were not through one way alone. Instead, they crosstalked with each other via many different pathways.

Although our work showed a better neuroprotective function of adjudin-preconditioned NSCs on I/R-induced brain injury, and adjudin may become a promising drug for clinical use that combines with stem cell-based therapy, further research is required before applying it to clinical research. The advantages of stem cell-based therapy are that grafted cells are not only able to secrete a plethora of soluble molecules to modulate the activation of host microglia/macrophages, thus modifying the release of inflammatory mediators and inhibiting oxidative stress, and thereby stabilizing the BBB, but are also capable of directly increasing cell proliferation within the SVZ, potentiating neuroblast migration, augmenting peri-ischemic angiogenesis, positively affecting the differentiation of endogenous neuroblasts and plasticity within the ischemic tissue. In addition, they could directly differentiate into postmitotic neurons, astrocytes, or oligodendrocytes to establish new neural circuits, and finally attenuate ischemic brain injury and improve neurobehavioral recovery [13, 15]. In order to examine whether adjudin preconditioning could achieve a better therapeutic effect and promote the transformation of adjudin to clinical use, more long-term experiments should be carried out. In this study we have included a 35-day study that has already shown encouraging results, but limitations also exist. Previous study showed that NSCs could survive and differentiate into functional neurons, attenuate infarction, and improve neurobehavioral recovery after stroke [91]. To further confirm the role of adjudin and study the mechanism of adjudin-pretreated NSCs in protecting the brain from ischemia injury, long-term experiments aiming to observe the number, localization, and differentiation status of transplanted cells in the ischemic brain are needed in future studies. Furthermore, adjudin has been demonstrated to have no apparent side effects in treated animals [33], but long-term safety remains a concern for clinical use when combined with cell sources. Although Lindvall and Kokaia [92] have demonstrated that no tumors were detected in five patients with Batten disease 2 years after transplantation of human fetal NSCs, the harsh microenvironment after I/R brain injury might influence tumorigenesis and differentiation profiles of grafted NSCs [93]. More observations in larger cohorts will be required for confirmation in the near future, with more definite conclusions regarding the safety of stem cell treatment to be made.

## Conclusion

In summary, our study demonstrated that adjudin preconditioning promoted NSC survival under H_2_O_2_ stimulation in vitro, reprogrammed NSCs to tolerate neuroinflammation and oxidative stress, and expressed higher levels of neurotrophic factors, resulting in augmenting the therapeutic efficiency of NSCs in transient focal ischemia in vivo. The protective effect of adjudin was achieved through activating the Akt pathway and inhibiting the p-p38 and p-JNK MAPK pathway. The beneficial effects of adjudin preconditioning may represent a safe approach for future clinical applications.

## Additional files

Additional file 1: Figure S1. The effect of adjudin on differentiation and proliferation of NSCs. Fluorescent photomicrographs indicate that the two concentrations of adjudin-pretreated NSCs were Nestin^+^, SOX2^+^, DCX^–^, GFAP^–^ for 10 μM and GFAP^+^ for 30 μM pretreated NSCs (**a**). Nuclei stained with DAPI. Scale bar = 100 μm. Role of adjuidin in NSC proliferation detected by immunostaining of Ki67 (**b**). Nuclei stained with DAPI. Scale bar = 100 μm (PNG 5980 kb)

Additional file 2: Figure S2. Cell viability of NSCs after pretreatment by adjudin and stimulated by H_2_O_2_ in vitro. Cell death and cell survival measured by LDH (**a**) and CCK-8 assay (**b**) after pretreatment with indicated concentrations of adjudin for 24 hours. Cell death and cell survival measured by LDH (**c**) and CCK-8 assay (**d**) after exposure to various concentrations of H_2_O_2_ (mM) for 1 hour. Bars represent mean ± SEM from three independent experiments. *P < 0.05, ***P < 0.001 (PNG 176 kb)

Additional file 3: Figure S3. Expression of antioxidant genes in NSCs with adjudin preconditioning in vitro. GCLC mRNA expression in adjudin-pretreated NSCs after H_2_O_2_ stimulation (**a**). mRNA expression levels of NOX4, HO-1, NQO1, and Nrf2 in adjudin-pretreated NSCs after H_2_O_2_ stimulation (**b–e**). Bars represent mean ± SEM from three independent experiments. *P < 0.05 (PNG 220 kb)

Additional file 4: Figure S4. Adjudin-pretreated NSCs inhibited phosphorylation of p-38 and JNK in vitro. Changes in p-p38, p-JNK, and p-ERK levels after 0.1 mM H_2_O_2_ stimulation in vitro. Representative western blot analysis showed phosphorylation levels of p38, JNK, and ERK in adjudin-pretreated NSCs which were stimulated by 0.1 mM H_2_O_2_ (**a, c, e**). Quantification of densitometric value of the protein bands normalized to total p38, JNK, and ERK1/2 (**b, d, f**). Bars represent mean ± SEM from three independent experiments. **P < 0.01, ***P < 0.001 (PNG 359 kb)

Additional file 5: Figure S5. Adjudin-pretreated NSCs inhibited phosphorylation of p65 in vitro. NSCs were pretreated with adjudin for 24 hours and then stimulated with H_2_O_2_ for 1 hour. Cell lysates were analyzed by western blot analysis with antibodies specific to phospho-p65 and p65 (PNG 116 kb)

## Abbreviations

AIS: Acute ischemic stroke
BBB: Blood–brain barrier
BCA: Bicinchoninic acid
BDNF: Brain-derived neurotrophic factor
BEC: Brain endothelial cells
CCA: Common carotid artery
CNS: Central nervous system
DAPI: 4,6-Diamidino-2-phenylindole
EB: Evans blue
ECA: External carotid artery
ECL: Enhanced chemiluminescence
EPC: Endothelial progenitor cell
ESC: Embryonic stem cell
GDNF: Glial cell-derived neurotrophic factor
GFP: Green fluorescent protein
I/R: Ischemia/reperfusion
ICA: Internal carotid artery
iPSC: Induced pluripotent stem cell
LDH: Lactate dehydrogenase
LPS: Lipopolysaccharide
MAPK: Mitogen-activated protein kinase
MCA: Middle cerebral artery
mNSS: Modified neurologic severity scores system
MSC: Mesenchymal stem cell
NGF: Nerve growth factor
NO: Nitric oxide
NSC: Neural stem cell
OPC: Oligodendrocyte progenitor cell
PBS: Phosphate-buffered saline
PCR: Polymerase chain reaction
PFA: Paraformaldehyde
PGE2: Prostaglandin E2
PMSF: Phenylmethylsulfonylfluoride
ROS: Oxygen species
TJ: Tight junction
tMCAO: Transient middle cerebral artery occlusion
tPA: Tissue plasminogen activator
