# Supplementary figures and images for: Adjudin-preconditioned neural stem cells enhance neuroprotection after ischemia reperfusion in mice
Source: Stem Cell Res Ther. 2017 Nov 7;8:248. doi: 10.1186/s13287-017-0677-0 (PMC5678778; doi:10.1186/s13287-017-0677-0)

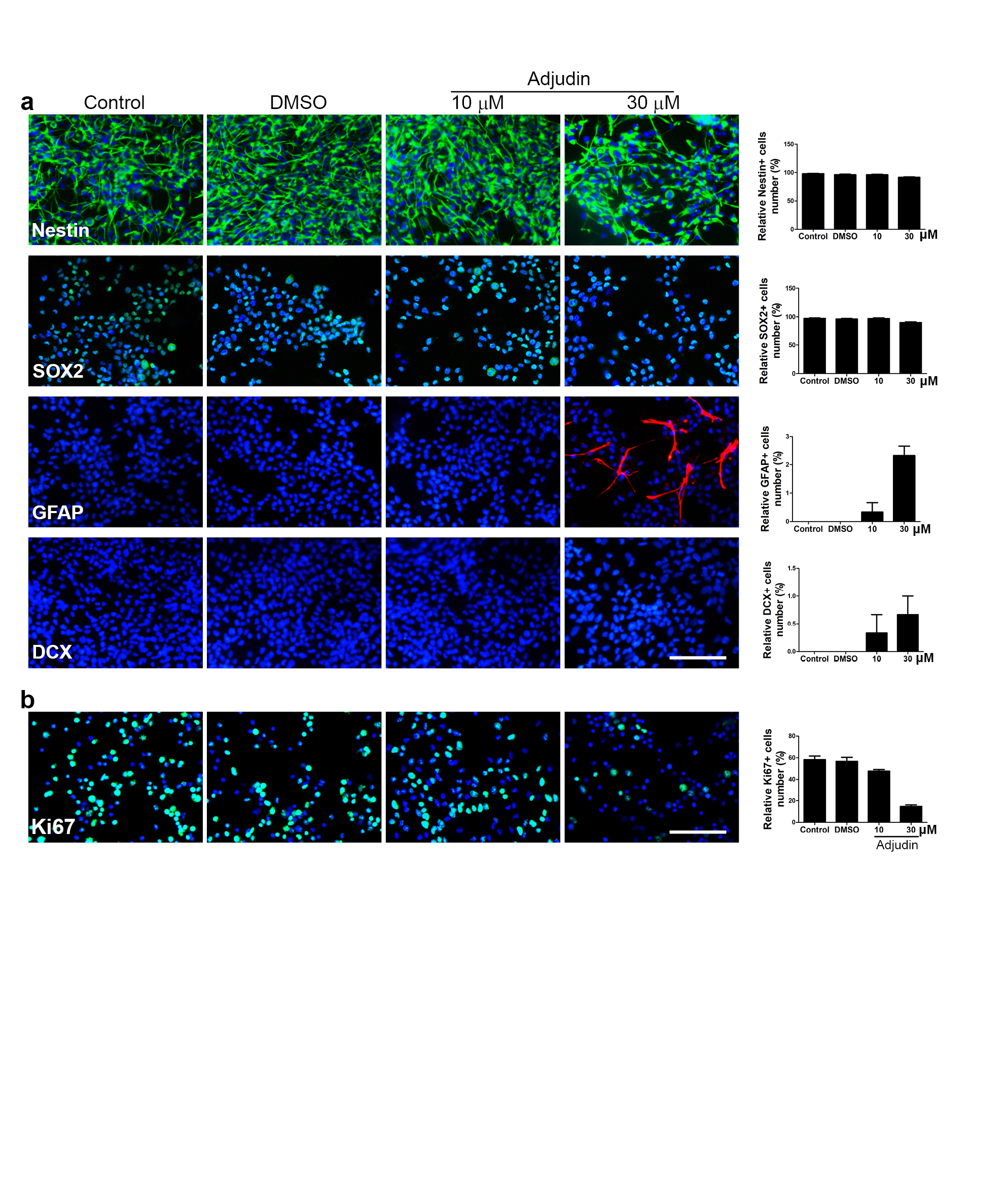

Supplement: Supplementary file 1 — The effect of adjudin on differentiation and proliferation of NSCs. Fluorescent photomicrographs indicate that the two concentrations of adjudin-pretreated NSCs were Nestin+, SOX2+, DCX–, GFAP– for 10 μM and GFAP+ for 30 μM pretreated NSCs (a). Nuclei stained with DAPI. Scale bar = 100 μm. Role of adjuidin in NSC proliferation detected by immunostaining of Ki67 (b). Nuclei stained with DAPI. Scale bar = 100 μm (PNG 5980 kb) [file 13287_2017_677_MOESM1_ESM.png]

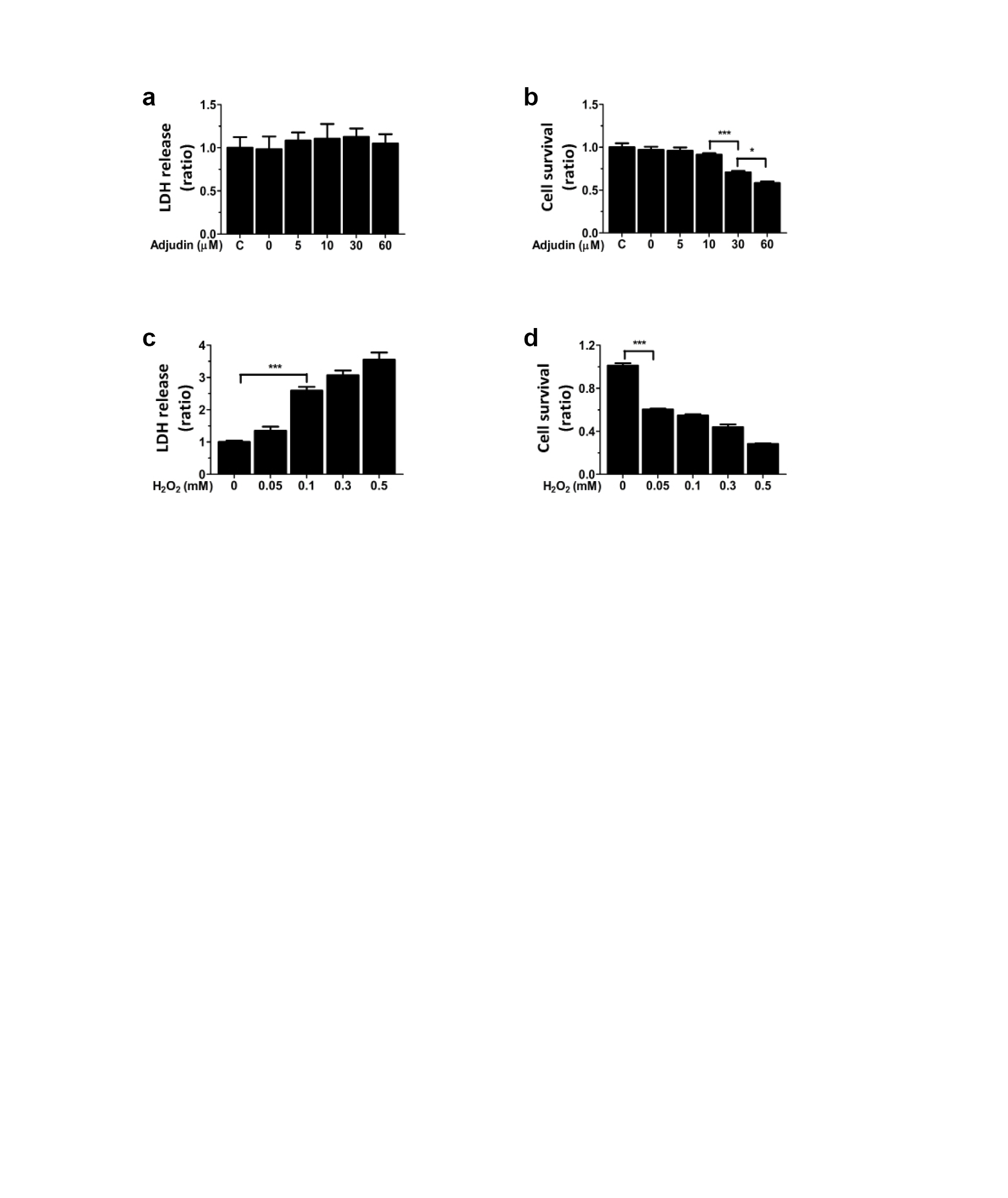

Supplement: Supplementary file 2 — Cell viability of NSCs after pretreatment by adjudin and stimulated by H2O2 in vitro. Cell death and cell survival measured by LDH (a) and CCK-8 assay (b) after pretreatment with indicated concentrations of adjudin for 24 hours. Cell death and cell survival measured by LDH (c) and CCK-8 assay (d) after exposure to various concentrations of H2O2 (mM) for 1 hour. Bars represent mean ± SEM from three independent experiments. *P < 0.05, ***P < 0.001 (PNG 176 kb) [file 13287_2017_677_MOESM2_ESM.png]

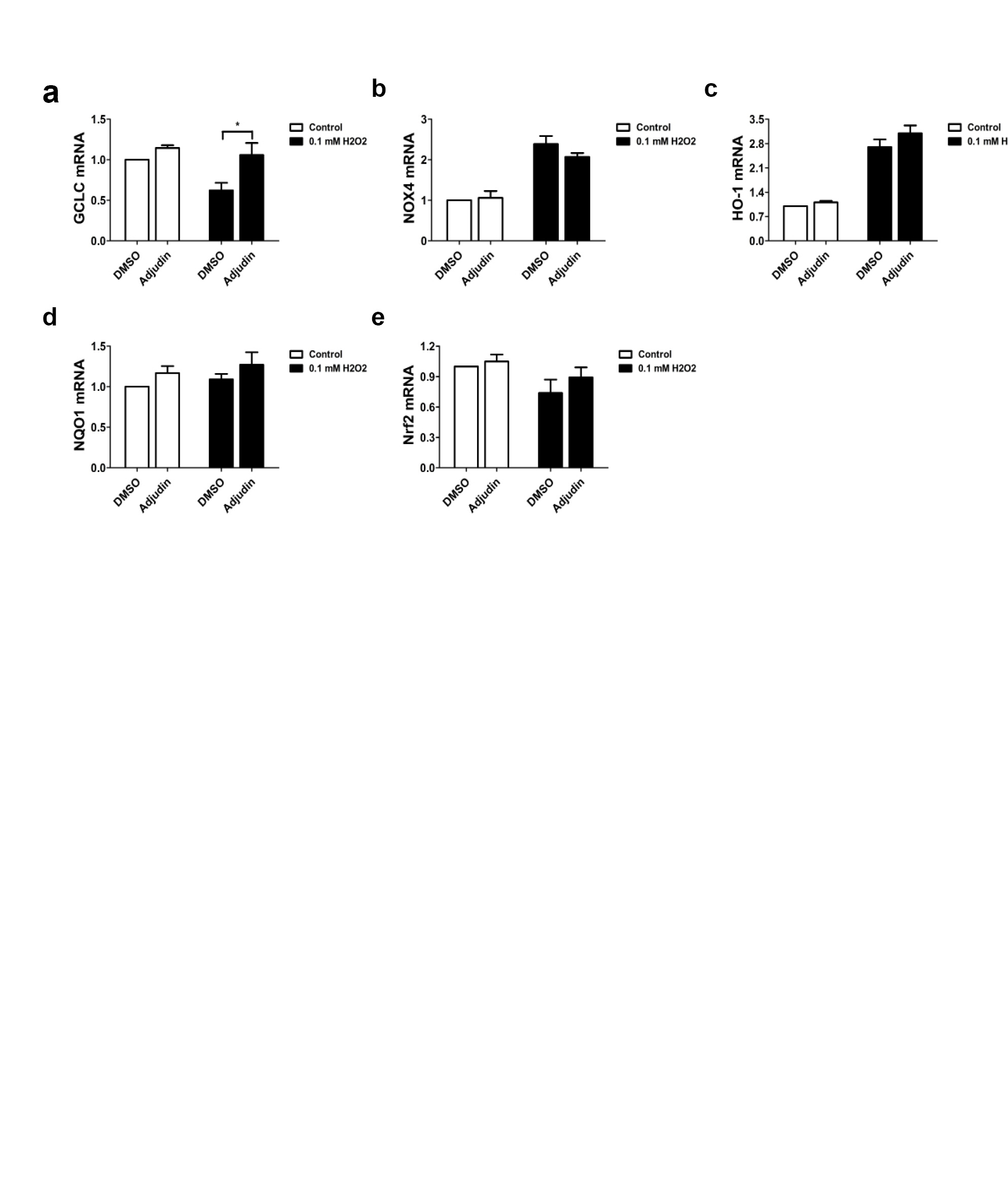

Supplement: Supplementary file 3 — Expression of antioxidant genes in NSCs with adjudin preconditioning in vitro. GCLC mRNA expression in adjudin-pretreated NSCs after H2O2 stimulation (a). mRNA expression levels of NOX4, HO-1, NQO1, and Nrf2 in adjudin-pretreated NSCs after H2O2 stimulation (b–e). Bars represent mean ± SEM from three independent experiments. *P < 0.05 (PNG 220 kb) [file 13287_2017_677_MOESM3_ESM.png]

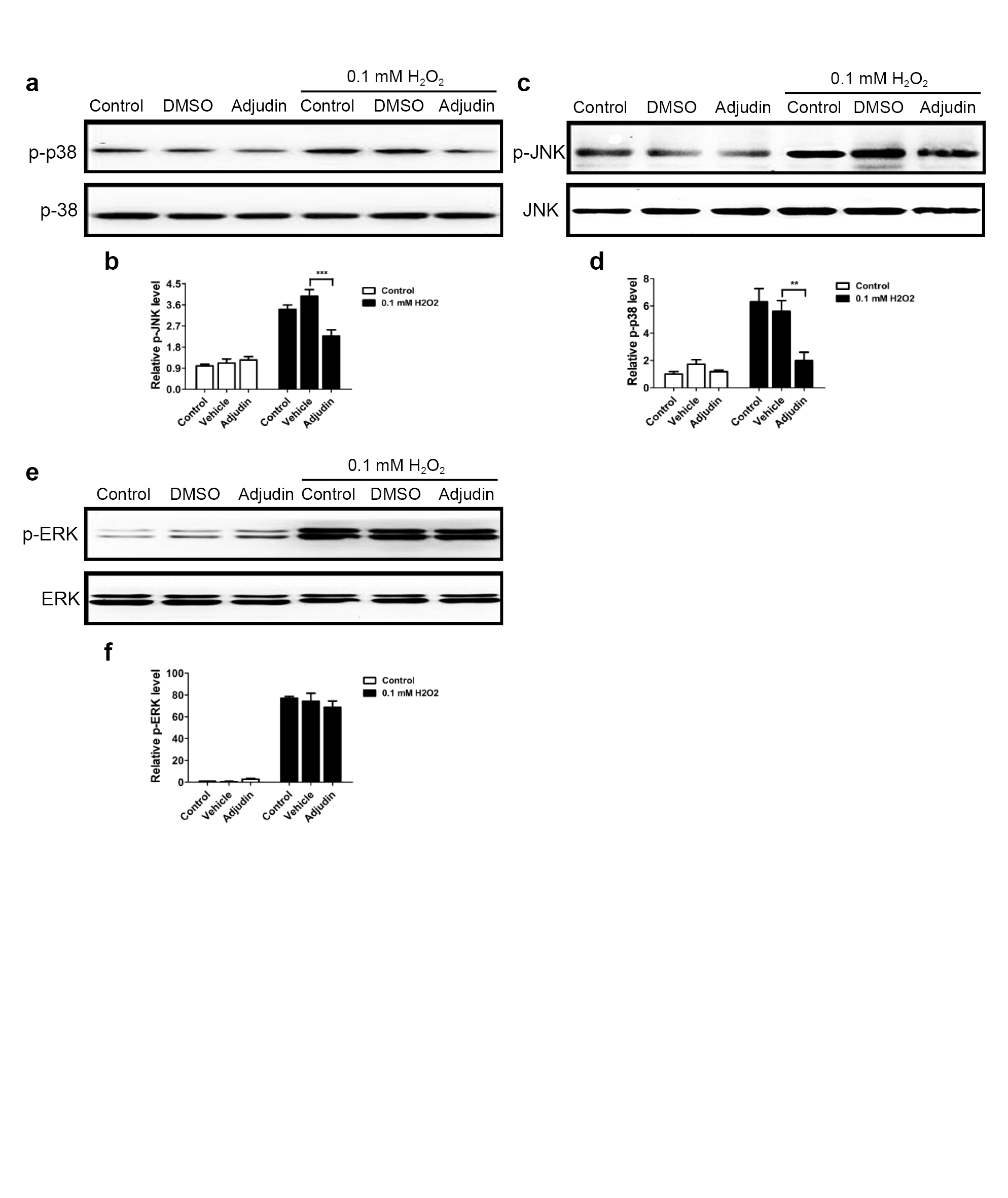

Supplement: Supplementary file 4 — Adjudin-pretreated NSCs inhibited phosphorylation of p-38 and JNK in vitro. Changes in p-p38, p-JNK, and p-ERK levels after 0.1 mM H2O2 stimulation in vitro. Representative western blot analysis showed phosphorylation levels of p38, JNK, and ERK in adjudin-pretreated NSCs which were stimulated by 0.1 mM H2O2 (a, c, e). Quantification of densitometric value of the protein bands normalized to total p38, JNK, and ERK1/2 (b, d, f). Bars represent mean ± SEM from three independent experiments. **P < 0.01, ***P < 0.001 (PNG 359 kb) [file 13287_2017_677_MOESM4_ESM.png]

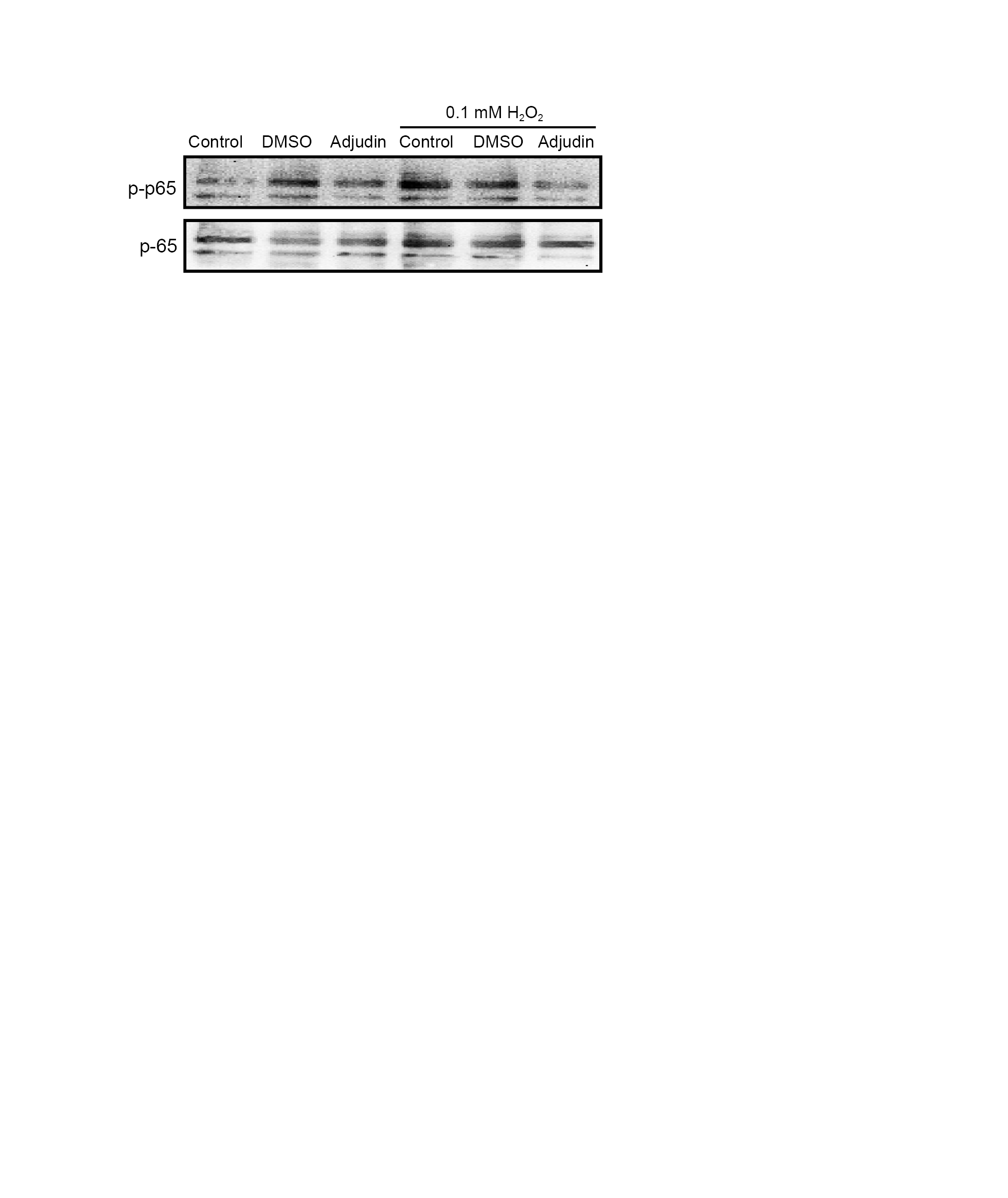

Supplement: Supplementary file 5 — Adjudin-pretreated NSCs inhibited phosphorylation of p65 in vitro. NSCs were pretreated with adjudin for 24 hours and then stimulated with H2O2 for 1 hour. Cell lysates were analyzed by western blot analysis with antibodies specific to phospho-p65 and p65 (PNG 116 kb) [file 13287_2017_677_MOESM5_ESM.png]
